# A Medical Student-Delivered Smoking Prevention Program, Education Against Tobacco, for Secondary Schools in Germany: Randomized Controlled Trial

**DOI:** 10.2196/jmir.7906

**Published:** 2017-06-06

**Authors:** Titus Josef Brinker, Andreas Dawid Owczarek, Werner Seeger, David Alexander Groneberg, Christian Martin Brieske, Philipp Jansen, Joachim Klode, Ingo Stoffels, Dirk Schadendorf, Benjamin Izar, Fabian Norbert Fries, Felix Johannes Hofmann

**Affiliations:** ^1^ Universities of Giessen and Marburg Lung Center; Member of the German Center for Lung Research (DZL) Department of Internal Medicine Justus-Liebig-University of Giessen Giessen Germany; ^2^ Institute of Occupational Medicine, Social Medicine and Environmental Medicine Goethe-University Frankfurt am Main Germany; ^3^ Essen University Hospital Department of Dermatology, Venerology and Allergology University of Duisburg-Essen Essen Germany; ^4^ West German Cancer Center University Duisburg-Essen Essen Germany; ^5^ German Cancer Consortium (DKTK) University of Heidelberg Heidelberg Germany; ^6^ Dana-Farber Cancer Institute Department of Medical Oncology Boston, MA United States; ^7^ Saarland University Medical Center and Saarland University Faculty of Medicine Department of Ophthalmology University of Homburg Homburg Germany

**Keywords:** medical students, tobacco prevention, secondary schools, smoking cessation, adolescents, school-based prevention

## Abstract

**Background:**

More than 8.5 million Germans suffer from chronic diseases attributable to smoking. Education Against Tobacco (EAT) is a multinational network of medical students who volunteer for school-based prevention in the classroom setting, amongst other activities. EAT has been implemented in 28 medical schools in Germany and is present in 13 additional countries around the globe. A recent quasi-experimental study showed significant short-term smoking cessation effects on 11-to-15-year-old adolescents.

**Objective:**

The aim of this study was to provide the first randomized long-term evaluation of the optimized 2014 EAT curriculum involving a photoaging software for its effectiveness in reducing the smoking prevalence among 11-to-15-year-old pupils in German secondary schools.

**Methods:**

A randomized controlled trial was undertaken with 1504 adolescents from 9 German secondary schools, aged 11-15 years in grades 6-8, of which 718 (47.74%) were identifiable for the prospective sample at the 12-month follow-up. The experimental study design included measurements at baseline (t1), 6 months (t2), and 12 months postintervention (t3), via questionnaire. The study groups consisted of 40 randomized classes that received the standardized EAT intervention (two medical student-led interactive modules taking 120 minutes total) and 34 control classes within the same schools (no intervention). The primary endpoint was the difference in smoking prevalence from t1 to t3 in the control group versus the difference from t1 to t3 in the intervention group. The differences in smoking behavior (smoking onset, quitting) between the two groups, as well as gender-specific effects, were studied as secondary outcomes.

**Results:**

None of the effects were significant due to a high loss-to-follow-up effect (52.26%, 786/1504). From baseline to the two follow-up time points, the prevalence of smoking increased from 3.1% to 5.2% to 7.2% in the control group and from 3.0% to 5.4% to 5.8% in the intervention group (number needed to treat [NNT]=68). Notable differences were observed between the groups for the female gender (4.2% to 9.5% for control vs 4.0% to 5.2% for intervention; NNT=24 for females vs NNT=207 for males), low educational background (7.3% to 12% for control vs 6.1% to 8.7% for intervention; NNT=30), and migrational background (students who claimed that at least one parent was not born in Germany) at the 12-month follow-up. The intervention appears to prevent smoking onset (NNT=63) but does not appear to initiate quitting.

**Conclusions:**

The intervention appears to prevent smoking, especially in females and students with a low educational background.

## Introduction

Most smokers start smoking during their early adolescence with the idea that smoking is glamorous; the problems related to vascular disease, lung cancer, chronic pulmonary disease and cataracts are too far in the future to fathom [1]. After multiple failed quit attempts, however, many smokers end up with tobacco-related diseases that physicians are unable to cure [2]. Despite the fact that effectiveness of inpatient smoking cessation was demonstrated in major trials [3] and was implemented in guidelines of almost all medical specialties [4], research has shown that physicians in Germany lack both the motivation (eg, role incongruence as a major barrier [5,6]) and education to deliver smoking cessation advice [5-8], especially before the onset of chronic disease [6]. The issue of undertreatment of tobacco use by physicians is known on a global scale [9,10]. It is estimated that global mortality attributable to tobacco will double from 5 million (2010) to 10 million per year in the coming decades [2].

Education Against Tobacco (EAT) is a multinational network of medical students that aims to provide science-based tobacco prevention to a large number of adolescents, and also sensitize prospective physicians to the importance of inpatient smoking cessation [11,12]. The network currently involves approximately 80 medical schools in 14 countries, with 1500 medical students educating more than 40,000 secondary school students in the classroom setting per year, while using and optimizing apps and strategies [13-16]. Since its foundation in Germany in 2012, the network has also instructed science-based smoking cessation curricula in 13 medical schools in Germany (of the 28 medical schools in Germany that take part in EAT) that are currently prospectively evaluating their effectiveness in preparing medical students for inpatient smoking cessation [7]. The two free science-based quit apps of EAT (*Smokerface* and *Smokerstop*) are downloaded more than a 1000 times per day and have been translated into most commonly spoken languages [14,15].

The 2016 HBSC international report by the WHO revealed that 13% of German boys and 15% of German girls at 15 years of age smoke cigarettes at least once a week [17]. Despite the decline in adolescent smoking over the last two decades, prevalence in Germany is still high and strong socioeconomic differences exist [17-19].

### Current Knowledge of School-Based Tobacco Prevention

Most school-based tobacco prevention curricula are ineffective and the evaluation of new curricula is mandatory [20]. A recently published evaluation of a short student and student-parent program from Germany did not show significant effectiveness among 7th grade students (7.6% and 7% prevalence in intervention groups vs 10.1% control group) at a 24-month follow-up. However, this result was mostly due to a too low sample size: only 47 schools were randomized due to an underestimated intracluster correlation coefficient [21,22]. The largest tobacco prevention program for secondary schools in Germany, the Smoke-Free Class Competition, has demonstrated limited effectiveness in increasing knowledge and making students initiate a quit attempt, but was not able to prevent smoking onset [23-25].

Physician-based programs relying on fear-inducing statements show no overall long-term effectiveness in reducing smoking prevalence [26-29], while limited new evidence suggests that asking questions about health consequences (rather than making statements) might be more effective to motivate current smokers to make a quit attempt [30].

A physician-based multimodal program in Berlin was evaluated in a quasi-experimental study, and showed significant short-term effects in preventing smoking onset, which might be a promising alternative to the traditional fear-based approaches of physician programs [31]. Outside of schools, a systematic review of inpatient physician-based smoking prevention and cessation programs for adolescents revealed that behavioral interventions show overall effectiveness in primary care [32].

### Previous Research on Education Against Tobacco

The effectiveness of an old version of the EAT curriculum on reducing smoking prevalence among German adolescents has only been investigated with a quasi-experimental design that contained potential sources of bias [11,12]. However, the study showed a significant association for reducing the smoking prevalence of secondary school students in Germany at 6-month follow-up by motivating them to make a quit attempt (n=1474 students). After this first evaluation, the curriculum was optimized for students with a lower educational level by using cognitive interviewing (we asked the students after the interventions what they found most convincing). The curriculum received more age-appropriate content, was optimized to be more interactive and gain-framed [33], and was equipped with a photoaging software [11,14].

### Introduction of the Education Against Tobacco App Smokerface

Photoaging desktop programs, in which an image is altered to predict future appearance, were effective in motivating 14-to-18-year-old females to quit smoking and increased the rate of quit attempts in 18-to-30-year-old young adults of both genders by 21% [34,35]. The broad availability of smartphones and adolescents' interest in their appearance [36] were harnessed to create the free 3D-photoaging smartphone app *Smokerface* [15] which animates the users’ selfies and reacts to touch (Multimedia Appendix 1). This app is downloaded 200 times per day and the current version of the app has a rating of 4.2/5 stars in the Playstore (Android, USA). This app was implemented via a poster-campaign in German secondary schools and is currently being evaluated in a large multicentered trial [14,37]. In 2014, the EAT curriculum was only available as a software program that was run on notebook computers, which captured participant’s faces via webcams.

The aim of this study was to provide the first randomized long-term evaluation of the optimized 2014 EAT curriculum involving a photoaging software for its effectiveness in reducing the smoking prevalence among 11-to-15-year-old pupils in German secondary schools.

## Methods

### Design

**Figure 1:**
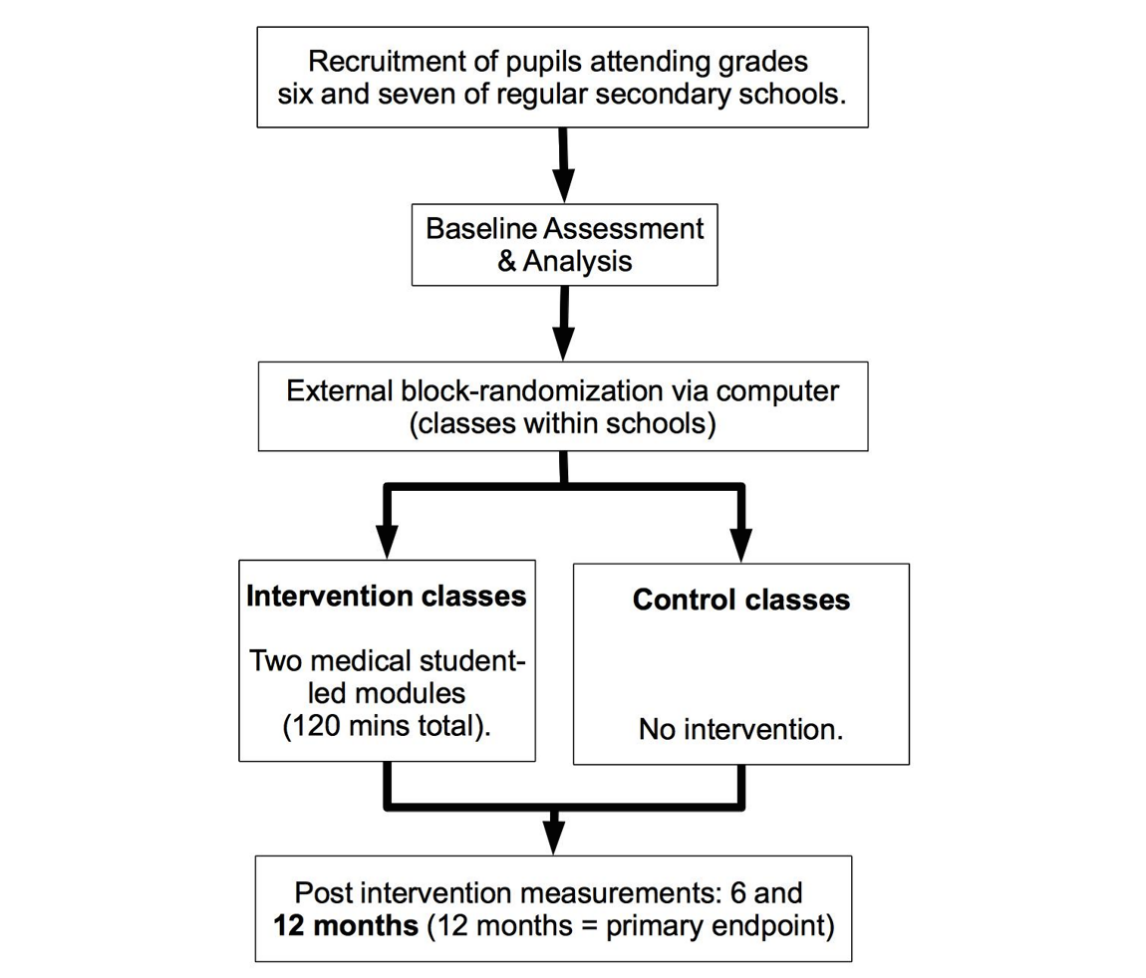
Study design.

The survey was designed as a randomized controlled trial with three measurements (baseline, 6 months, and 12 months postintervention) [12]. The study period was October 2014 until March 2016. Participants in the two study groups (intervention and control) were questioned up to 2 weeks in advance of the intervention (t1), and 6 months (t2) and 12 months (t3) thereafter (Figure 1).

### Randomization

In accordance with the recommendations of the Cochrane Analysis [20], randomization was externally and centrally performed by a statistician from the University of Frankfurt, Germany on the class level within each school via block randomization. Due to the fact that the statistician performed the randomization one school at a time, there was a slight imbalance between groups (40 intervention and 34 control classes).

#### Participants

A total of 1504 eligible secondary school students from 74 classes (from 9 eligible schools) entered baseline data. All participants fulfilled the inclusion criteria. Students aged 10-15 years attending grades 6-8 of a secondary general, intermediate, grammar, or comprehensive school were eligible [12]. Baseline data were collected from October 2014 to March 2015. Follow-up data were collected from April 2015 to March 2016. A total of 718 participants provided data at both time points (t1 and t3) that was used for primary endpoint analyses. The loss to follow-up effect was 52.26% (786/1504) due to problems with the identifier code (see Discussion section).

#### Attrition Analysis

The participants who dropped out at follow-up (t3) were analyzed with logistic regression analysis and showed no systematic bias regarding the interaction between: study group and smoking status (*P*=0.520), study group and gender (*P*=0.131), study group and age (*P*=0.427), or study group and school type (grammar school vs lower educational school type; *P*=0.440). However, there was a systematic bias regarding gender as a main effect (odds ratio [OR]=0.511, 95% CI 0.412-0.633, *P*<0.001) meaning that more boys dropped out than girls. There was a systematic difference between groups regarding school type. The interaction between study group (0=control and 1=intervention) and school type (0=no grammar school and 1=grammar school) was significant (OR=0.618, 95% CI 0.410-0.933, *P*=0.022). In the intervention group, the dropout was lower in grammar schools, whereas the dropouts were at similar levels in the control group (intervention group: grammar 37.9% [165/435], no grammar 55.0% [213/387]; control group: grammar 45.9% [174/379], no grammar 49.8% [151/303]). Dropouts include cases that could not be matched due to difficulties with the identifier code.

### Intervention

The intervention was the 2014 EAT curriculum, which consisted of two interactive 60-minute modules delivered by medical students from the University of Giessen. The medical students did not volunteer but had the duty to perform a school visit based on their participation in the EAT course. The app version of *Smokerface* was available at the time but was not advertised or mentioned to the students, to avoid contamination of the control group. The medical students received standardized training that lasted 45 minutes. Students were asked in advance to read through the classroom curriculum and then met with an experienced medical student who went through all key procedures of the intervention.

The first part of the intervention was presented by two-to-six medical students and a patient with a tobacco-related disease to all pupils in a large room within the school. The presentation consisted of a PowerPoint (Microsoft; Redmond, WA, USA) presentation which aimed at addressing age-appropriate topics in an interactive manner to help the students reframe a positive nonsmoking image, which included: physical performance (with the example of famous German soccer players), saved money, addiction versus freedom, examples of tobacco advertising and how the students would advertise tobacco products if given the opportunity, attractiveness, and photoaged images. At the end of each presentation, a patient with a tobacco-related disease (chronic obstructive pulmonary disease) was introduced and then interviewed by the medical students and pupils.

**Figure 2:**
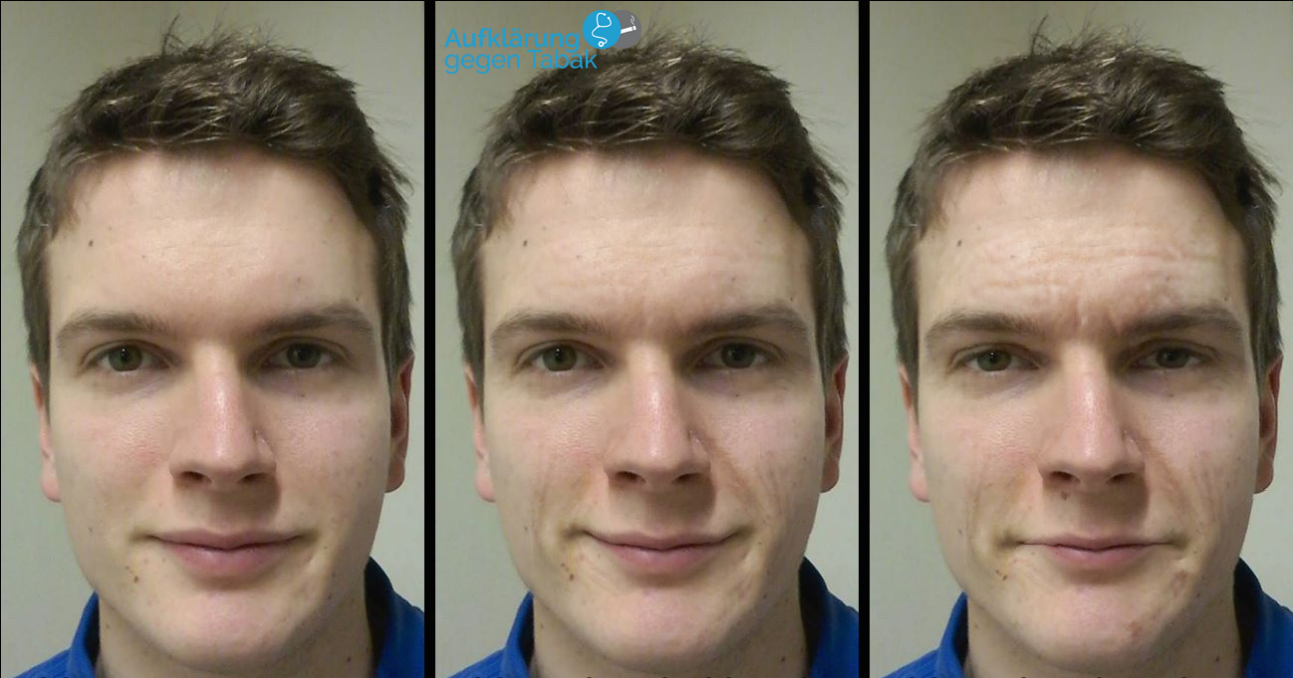
Effects of the first version of the Smokerface software used in the study; left image: original picture; middle: normal aging for 15 years; right: smoking a pack a day for 15 years.

The second part of the intervention consisted of a classroom seminar and a photoaging intervention for every individual pupil. Two medical students tutored one school class consisting of approximately 25 students in an interactive manner. While one medical student presented the curriculum, groups of approximately five pupils were sent to another room for the morphing procedure with an older version of the self-developed *Smokerface* software, which was in 2D (Figure 2) [15]. Topics included: skin damage, harm to the rest of the body, drop in physical performance, growth during adolescence [38], freedom and independence, advertising, and cost. The full 2014 classroom curriculum can be accessed online in English [13].

#### Photoaging Intervention

**Figure 3:**
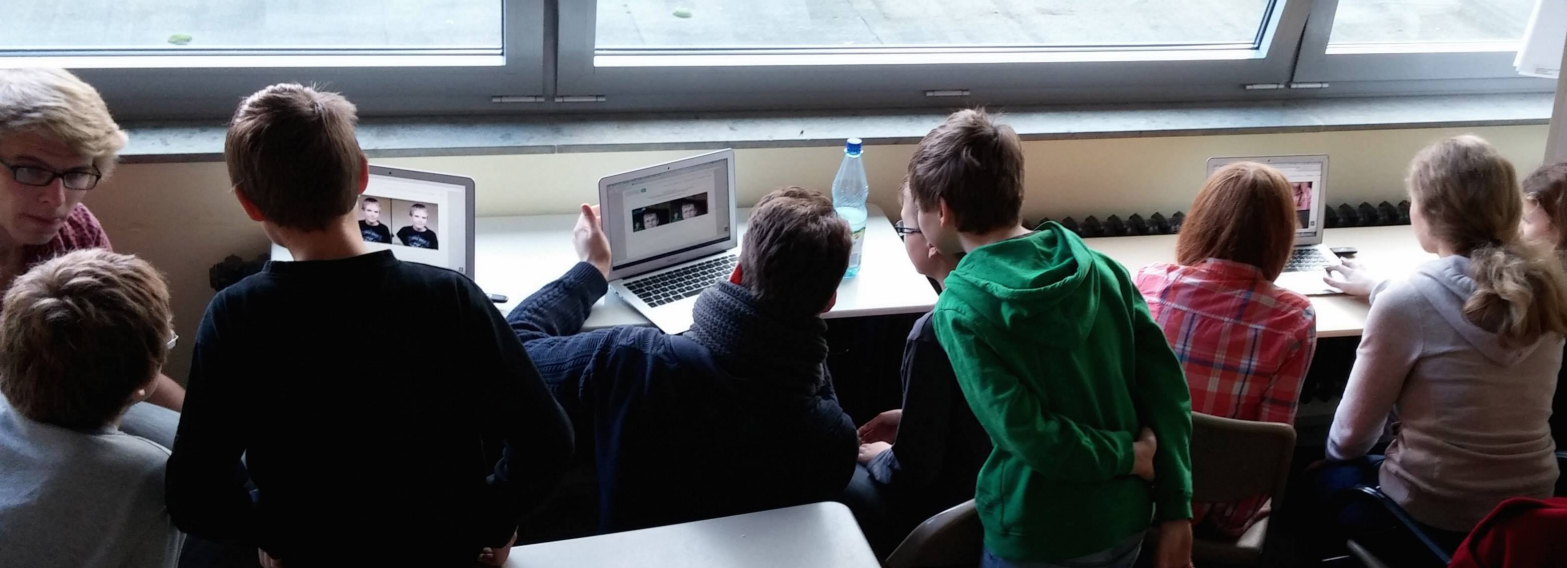
"Facemorphing room" hosted by three medical students in a school with three classes.

Four Macbook Air (Apple, California) computers were purchased to run the photoaging intervention in four classes simultaneously, and to make sure that every single pupil got their face photoaged. Wireless Local Area Network (W-LAN) sticks were also purchased, as many schools did not have W-LAN at the time but the app had to communicate with servers to perform the morphing process (the current version of the app runs offline). Every student received the photoaging intervention; groups of five pupils were sent out to an external room where one medical student per class was taking pictures with the webcam and then showed them the predicted result of smoking on their own faces (Figure 3). The pupils chose whether they kept their image strictly for themselves or shared it with friends. Each student got a brief explanation of the skin changes by the medical student, which was reinforced in the classroom.

### Data Collection

Data were collected via a published questionnaire that was used in the previous investigation of the same age group, which was optimized by cognitive interviewing [11,39]. All items were based on three established studies declared to be high quality by the recent Cochrane Analysis and were either used in their original form or adapted to the specific circumstances of the recent study [20,40-42]. In addition to sociodemographic data (age, gender, school type), the questionnaire captured the smoking status of the school students concerning e-cigarette, water-pipe, and cigarette consumption. The only alteration to the protocol was that data entry was not manually performed; a scanning software was used to make the process less time consuming. However, for this approach to work the identifier code was changed from letters and numbers to numbers only, which resulted in a large fraction of students that were not identifiable at follow-up.

### Outcomes

The primary endpoint was the difference in smoking prevalence from t1 to t3 in the control group versus the difference from t1 to t3 in the intervention group. The differences in smoking behavior (smoking onset, quit attempts) between the two groups, as well as gender-specific effects, were studied as secondary outcomes.

### Statistical Analyses

To examine baseline differences χ^2^tests, t-tests and Mann-Whitney U Tests were used (see Multimedia Appendix 2 for details). The effects of predictors (gender, culture, and social characteristics) on smoking behavior after 12 months (t3) were calculated by robust panel logistic regression analysis. The significance level was 5% for t-tests (double-sided) and 95% for CIs (double-sided). Statistical analyses were performed using SPSS Statistics Version 23 (IBM; Armong, USA) and STATA 14 (StataCorp; Texas, USA). The group allocation in the sample was based on the class level. To account for this clustering, statistically robust panel logistic regression was used (xtlogit procedure with vce [cluster] option). This procedure was also used to calculate the difference from t1 to t3 of the smoking prevalence in the control group versus the difference from t1 to t3 in the intervention group (the primary endpoint) by using STATA 14. The number needed to treat (NNT) calculation was performed without controlling for baseline differences, as these data were comparable due to randomization. The NNT was calculated for the total effect (preventing smoking onset and initiating quit attempts) and for quit attempts and smoking onset individually. Any NNT values higher than 200 were not reported due to lack of relevance.

### Legal Approval

In accordance with Good Epidemiologic Practice guidelines, an ethics waiver and all legal permissions were obtained from the responsible institutions before data collection started. Written informed consent was obtained from all students and their parents. Teachers had to be present during the intervention and school personnel in charge of possible adverse health events were present in each school visited. No adverse events occurred.

**Table 1:** Cigarette smoking prevalence at baseline (t1), 6 months (t2), and 12 months (t3).

	Time point	Intervention and Control Group	Intervention Group	Control Group	Number Needed to Treat
Total	t1	22/718 (3.1%)	12/400 (3.0%)	10/318 (3.1%)	
	t2	42/788 (5.3%)	23/425 (5.4%)	19/363 (5.2%)	
	t3	46/718 (6.4%)	23/400 (5.8%)	23/318 (7.2%)	68
Grammar schools	t1	4/446 (0.9%)	3/252 (1.2%)	1/194 (0.5%)	
	t2	8/473 (1.7%)	4/251 (1.6%)	4/222 (1.8%)	
	t3	17/406 (4.2%)	9/227 (4.0%)	8/179 (4.5%)	199
Lower educational background	t1	18/272 (6.6%)	9/148 (6.1%)	9/124 (7.3%)	
	t2	34/315 (10.8%)	19/174 (10.9%)	15/141 (10.6%)	
	t3	28/275 (10.2%)	13/150 (8.7%)	15/125 (12%)	30
Male	t1	6/280 (2.1%)	3/146 (2.1%)	3/134 (2.2%)	
	t2	9/325 (2.8%)	4/168 (2.4%)	5/157 (3.2%)	
	t3	12/264 (4.5%)	6/139 (4.3%)	6/125 (4.8%)	207
Female	t1	16/392 (4.1%)	9/226 (4.0%)	7/166 (4.2%)	
	t2	28/392 (7.1%)	16/216 (7.4%)	12/176 (6.8%)	
	t3	27/387 (7.0%)	12/229 (5.2%)	15/158 (9.5%)	24
Migrational background: no	t1	15/480 (3.1%)	7/264 (2.7%)	8/216 (3.7%)	
	t2	24/507 (4.7%)	13/259 (5.0%)	11/248 (4.4%)	
	t3	31/476 (6.5%)	15/263 (5.7%)	16/213 (7.5%)	56
Migrational background: yes	t1	6/211 (2.8%)	4/123 (3.3%)	2/88 (2.3%)	
	t2	13/231 (5.6%)	9/136 (6.6%)	4/95 (4.2%)	
	t3	14/197 (7.1%)	7/114 (6.1%)	7/83 (8.4%)	44

## Results

### Baseline Data

The mean age of the 1504 eligible participants at baseline (Multimedia Appendix 2) was 12.4 years (range 11-15 years) and 49.17% (681/1385) were female. Of the participants, 54.12% (814/1504) attended grammar schools and the remaining 45.88% (690/1504) attended comprehensive schools (which were classified in the survey as *lower education level*). The survey identified 3.89% (55/1414) of participants as cigarette smokers at baseline. There were no significant differences concerning the number of cigarette smokers in each group (*P*=0.797; Multimedia Appendix 2).

### Follow-Up Data

Analyses of the data were based on the originally assigned groups (see Table 1). There were 400 pupils in the intervention group and 318 pupils in the control group who had participated in the survey at both relevant time points that could be identified (baseline sample=1504; prospective sample=718 pupils; loss to follow-up=786 pupils).

None of the effects were significant due to a high loss-to-follow-up effect (52.26%, 786/1504) including the primary endpoint. From baseline to the two follow-up time points, the smoking prevalence increased from 3.1% to 5.2% to 7.2% in the control group and from 3% to 5.4% to 5.8% in the intervention group (NNT=68) with notable nonsystematic effects for the female gender (4.2% to 9.5% for control vs 4% to 5.2% for intervention; NNT=24 for females vs NNT=207 for males), low educational background (7.3% to 12% for control vs 6.1% to 8.7% for intervention; NNT=30), and migrational background (students who claimed that at least one parent was not born in Germany) at 12-month follow-up. The intervention appears to prevent smoking onset (NNT=63) but does not appear to initiate quit attempts. Details on smoking prevalence among subgroups can be found in Table 1 (including NNT).

### Primary Endpoint

There was no significant effect for the defined primary endpoint (OR=0.74; 95% CI 0.21-2.56; *P*=0.63) calculated with the prospective sample of 718 participants (Table 2). The percentage of students who claimed to be smokers increased from 3.1% (t1) to 7.2% (t3) in the control group, but the increase was less dramatic in the intervention group (3% [t1] to 5.4% [t3]).

### Secondary Outcomes

At the 12-month follow-up, 17 of 318 control group students (5.3%) had started smoking but only 15 of 400 intervention group students had started smoking (3.8%; Table 3). No secondary preventive effects (quit attempts) were noted.

**Table 2:** Primary endpoint calculated by robust panel logistic regression (xtlogit procedure with vce [cluster] option). Difference in smoking prevalence from t1 to t3 of the smoking prevalence in the control group versus the difference from t1 to t3 in the intervention group (see Methods section).

	Odds Ratio	Standard Error	*P*-value	Lower-CI	Upper-CI
Complete sample, strongly balanced (n=718)	0.74	0.47	0.630	0.21	2.56
Only nongrammar schools (n=272)	0.72	0.45	0.606	0.21	2.47
Only female students (n=417)	0.44	0.40	0.368	0.08	2.60
Only students with migration background (n=206)	0.84	0.74	0.848	0.15	4.76

**Table 3:** Nominal and percentage effects of the intervention on the smoking status (secondary outcomes, from t1 to t3).

	Prospective smoking status (t1-t3)			
	Remains nonsmoker	Commences smoking	Ceases smoking	Remains smoker
Control Group, n (%)	291 (91.5)	17 (5.3)	4 (1.3)	6 (1.9)
Intervention Group, n (%)	373 (93.2)	15 (3.8)	4 (1.0)	9 (2.0)
Total, N	664	32	8	14

## Discussion

This is the first long-term evaluation of a photoaging intervention to prevent smoking and the first completed randomized trial on medical-student-delivered school-based tobacco prevention to date [16]. The present study suggests that photoaging is effective at preventing smoking onset, especially in female students (NNT=24) and students with a low educational (NNT=30) or migrational backgrounds (NNT=44).

### Interpretation

Available cross-sectional data reveals that photoaging interventions are effective in motivating 14-18-year-old female smokers to make a quit attempt [35], so it was hypothesized that secondary preventive effects would be present in the sample, which was not the case. However, the intervention showed a smaller NNT for females versus males in preventing smoking onset (NNT=24 for females vs 207 for males) and the uptake of smoking for females in the intervention group was lower after 6-month and 12-month follow-ups compared to males exposed to the intervention. These data reflect the findings of a recent study by Baudson et al that was conducted with 2950 German adolescents of both genders aged 10-19 years, which showed that self-concept of appearance is the strongest predictor for self-esteem, and that this is especially true for girls and adolescents from schools with a low educational level [36]. It is notable that our data shows an NNT of 30 for nongrammar schools but an NNT of 199 for grammar schools.

However, a recent study demonstrated that the theory of planned behavior needs to be taken into account when implementing photoaging in school settings with adolescents [14]. It is possible that the intervention could not show an effect on males due to a lack of group effects and elements that increase catamnesis (such as posters [37]), as other data also indicate that photoaging interventions are effective for both genders [14,34,43].

The implementation of cost-effective measures to prevent smoking in adolescents and, moreover, the sensitization of prospective physicians to tobacco-attributable diseases, tobacco prevention, and improved communication of these issues in medicine, is addressed by the program [5,9,44,45].

### Limitations

While the groups were successfully randomized and provided comparable baseline data, an unusually high loss-to-follow-up effect led to no significant results to report. However, the descriptive data collected at three different time points and effect size estimates, such as number needed to treat (which is widely accepted as an indicator for clinical relevance), allowed for data interpretation [46]. The photoaging software became available as an app during the study period and was advertised via television. Thus, a small portion of the control group might have been exposed to the photoaging intervention. In addition to this variable, intervention and control classes were in the same schools, which made it possible for the curriculum content to be exchanged. Furthermore, in some cases teachers would not adhere to the handouts pertaining to nondisclosure towards the control classes and disclose information on curriculum content. A follow-up study should use schools, but not classes, as a cluster.

Our study relied on self-reports obtained from adolescents via questionnaires that has been critically reviewed by Gorber et al in 2009, in which cotinine saliva testing was suggested [47]. However, Gorber et al did not take into account e-cigarettes, which are a limitation to cotinine saliva testing (because they also may contain nicotine). Additionally, saliva testing was prohibited by the ministry of cultural affairs in Germany, and because this is a randomized study, the influence of social desirability bias should have the same influence in both groups.

### Dissemination of the Intervention

Approximately 5 years after EAT was founded (January 2012), the program has more participating mentors (1500 medical students) and interactively educates more secondary school students per year (40,000) than any other known school-based physician-delivered or medical-student-delivered tobacco prevention program in Germany or, to our knowledge, worldwide. This program is currently present in over 80 medical schools in 14 countries. The apps used by the medical students in the classroom are freely available around the globe and have been translated into the six most commonly spoken languages worldwide.

### Conclusions

In conclusion, the 2014 EAT curriculum focusing on photoaging aspects of smoking appears to be most effective in females or students with a low educational background, but appears to lack effectiveness in grammar school students and male adolescents. Further research and long-term evaluation in sufficiently powered trials, as well as new ways of implementation, are needed to further evaluate and optimize our program.

Acknowledgements

We would like to thank the volunteer medical students Hannes Tabert, Svea Holtz, Stefan Henkel, Laura Schwab, Thorben Sämann, Andreas Owczarek, Felix Neumann, Anika Wolf, Dominik Penka, and Lorena Steinbach for their strong engagement within the EAT program, which made the relatively large sample size possible. This study was funded by the German Center for Lung Research (DZL).

## Appendix

Multimedia Appendix 1 Video 1 (touch effect of the Smokerface App).

Multimedia Appendix 2 Descriptive data at baseline.

Multimedia Appendix 3 CONSORT EHealth Checklist.

## Abbreviations

EAT: Education Against Tobacco
NNT: number needed to treat
OR: odds ratio
W-LAN: Wireless Local Area Network
