# Supplementary material for: A Medical Student-Delivered Smoking Prevention Program, Education Against Tobacco, for Secondary Schools in Germany: Randomized Controlled Trial
Source: J Med Internet Res. 2017 Jun 6;19(6):e199. doi: 10.2196/jmir.7906 (PMC5478798; doi:10.2196/jmir.7906)
Supplement: Multimedia Appendix 2 [file jmir_v19i6e199_app2.pdf]

Multimedia Appendix 1. Descriptive data at baseline.

Variable	Entire sample n/N (%)	Intervention group n/N (%)	Control group n/N (%)	p-value
**Number of pupils**	1504/1504 (100%)	822/1504 (54.7%)	682/1504 (45.3%)	<0.001[1]
**Number of classes**	74/74 (100%)	40/74 (54.1%)	34/74 (45.9%)	0.485[1]
**Grammar school students**	814/1504 (54.1%)	435/822 (52.9%)	379/682 (55.6%)	0.304[1]
**Gender**	1385/1504 (92.1%)			
Female	681/1385 (49.2 %)	378/748 (50.5%)	303/637 (47.6%)	0.271[1]
Male	704/1385 (50.8%)	370/748 (49.5%)	334/637 (52.4%)	
**Age**	1452/1504 (96.5%)	789/1452 (54.3%)	663/1452 (45.7%)	
		M=12.32 (SD=0.89)	M=12.40 (SD=0.86)	0.098[2]
**Both my parents and I were born in Germany (yes)**	949/1420 (66.8%)	511/776 (65.9%)	438/644 (68%)	0.389[1]
**Which tobacco products have you ever tried?**				
cigarettes	199/1492 (13.3%)	105/818 (12.8%)	94/674 (13.9%)	0.530[1]
e-cigarettes/e-shishas/e-	236/1492 (15.8%)	117/818 (14.3%)	119/674 (17.7%)	0.077[1]

cigar/e-pipe				
water pipe with tobacco	96/1492 (6.4%)	47/818 (5.7%)	49/674 (7.3%)	0.233[1]
cigar or cigarillo	34/1492 (2.3%)	17/818 (2.1%)	17/674 (2.5%)	0.567[1]
**Which tobacco products have you used at least once in the past 30 days? (=current use)**				
cigarettes	55/1414 (3.9%)	31/773 (4%)	24/641 (3.7%)	0.797[1]
e-cigarettes/e-shishas/e-cigar/e-pipe	54/1424 (3.8%)	29/787 (3.7%)	25/637 (3.9%)	0.814[1]
water pipe with tobacco	32/1437 (2.2%)	15/788 (1.9%)	17/649 (2.6%)	0.360[1]
cigar or cigarillo				
Pupils who currently used two products in the past 30 days (dual use)	39/1426 (2.7%)	22/790 (2.8%)	17/636 (2.7%)	0.898[1]
Dual use: e-cigarettes and cigarettes	22/1439 (1.5%)	14/793 (1.8%)	8/646 (1.2%)	0.418[1]
Dual use: waterpipe with tobacco and cigarettes	15/1442 (1%)	10/791 (1.3%)	5/651 (0.8%)	0.356[1]
**Current cigarette smokers**	55/1414 (3.9%)	31/773 (4%)	24/641 (3.7%)	0.797[1]

More than 10 cigarettes/day	7/49 (14.3%)	4/30 (13.3%)	3/19 (15.8%)	0.375[3]
7-10 cigarettes/day	9/49 (18.4%)	6/30 (20%)	3/19 (15.8%)	
4-6 cigarettes/day	7/49 (14.3%)	6/30 (20%)	1/19 (5.3%)	
1-3 cigarettes/day	2/49 (4.1%)	1/30 (3.3%)	1/19 (5.3%)	
1-6 cigarettes/week	2/49 (4.1%)	2/30 (6.7%)	0/19 (0%)	
At least once per month	11/49 (44.9%)	11/30 (36.7%)	11/19 (57.9%)	
**Current water pipe with tobacco smokers**	32/1437 (2.2%)	15/788 (1.9%)	17/649 (2.6%)	0.360[1]
**At least once daily**	1/31 (3.2%)	1/15 (6.7%)	0/16 (0%)	0.310[3]
**At least once per week**	9/31 (29%)	3/15 (20%)	6/16 (37.5%)	
**At least once per month**	14/31 (45.2%)	5/15 (40%)	8/16 (50%)	
**At least once per year**	7/31 (22.6%)	5/15 (33.3%)	2/16 (12.5%)	
**Current e-cigarette or e-shisha smokers**				
**At least once daily**	4/53 (7.5%)	4/29 (13.8%)	0/24 (0%)	0.909[3]

**At least once per week**	9/53 (17%)	4/29 (13.8%)	5/24 (20.8%)	
**At least once per month**	19/53 (35.8%)	8/29 (27.6%)	11/24 (45.8%)	
**At least once per year**	21/53 (39.6%)	13/29 (44.8%)	8/24 (33.3%)	
**Either mother or father currently smoke**	632/1490 (42.4%)	341/813 (41.9%)	291/677 (43%)	0.686[1]

[1] Chi-Square-Test (N-1-Test for 2x2-tables)
[2] T-Test
[3] Mann-Whitney-U-Test, corrected for bindings
