# Supplementary material for: A Medical Student-Delivered Smoking Prevention Program, Education Against Tobacco, for Secondary Schools in Germany: Randomized Controlled Trial
Source: J Med Internet Res. 2017 Jun 6;19(6):e199. doi: 10.2196/jmir.7906 (PMC5478798; doi:10.2196/jmir.7906)
Supplement: Multimedia Appendix 3 [file jmir_v19i6e199_app3.pdf]

CONSORT-EHEALTH (V 1.6.1) - Submission/Publication Form

The CONSORT-EHEALTH checklist is intended for authors of randomized trials evaluating web-based and Internet-based applications/interventions, including mobile interventions, electronic games (incl multiplayer games), social media, certain telehealth applications, and other interactive and/or networked electronic applications. Some of the items (e.g. all subitems under item 5 - description of the intervention) may also be applicable for other study designs.

The goal of the CONSORT EHEALTH checklist and guideline is to be
a) a guide for reporting for authors of RCTs,
b) to form a basis for appraisal of an ehealth trial (in terms of validity)

CONSORT-EHEALTH items/subitems are MANDATORY reporting items for studies published in the Journal of Medical Internet Research and other journals / scientific societies endorsing the checklist.

Items numbered 1., 2., 3., 4a., 4b etc are original CONSORT or CONSORT-NPT (non-pharmacologic treatment) items.
Items with Roman numerals (i., ii, iii, iv etc.) are CONSORT-EHEALTH extensions/clarifications.

As the CONSORT-EHEALTH checklist is still considered in a formative stage, we would ask that you also RATE ON A SCALE OF 1-5 how important/useful you feel each item is FOR THE PURPOSE OF THE CHECKLIST and reporting guideline (optional).

Mandatory reporting items are marked with a red *.
In the textboxes, either copy & paste the relevant sections from your manuscript into this form - please include any quotes from your manuscript in QUOTATION MARKS,
or answer directly by providing additional information not in the manuscript, or elaborating on why the item was not relevant for this study.

YOUR ANSWERS WILL BE PUBLISHED AS A SUPPLEMENTARY FILE TO YOUR PUBLICATION IN JMIR AND ARE CONSIDERED PART OF YOUR PUBLICATION (IF ACCEPTED).
Please fill in these questions diligently. Information will not be copyedited, so please use proper spelling and grammar, use correct capitalization, and avoid abbreviations.

DO NOT FORGET TO SAVE AS PDF _AND_ CLICK THE SUBMIT BUTTON SO YOUR ANSWERS ARE IN OUR DATABASE !!!

Citation Suggestion (if you append the pdf as Appendix we suggest to cite this paper in the caption):
Eysenbach G, CONSORT-EHEALTH Group
CONSORT-EHEALTH: Improving and Standardizing Evaluation Reports of Web-based and Mobile Health Interventions
J Med Internet Res 2011;13(4):e126
URL: http://www.jmir.org/2011/4/e126/
doi: 10.2196/jmir.1923
PMID: 22209829

* Erforderlich

Ihre Antwort übersteigt die maximale Zeichenanzahl. Kürzen Sie einige Sätze in Ihrer Antwort.

Your name *
First Last

Titus Brinker

Primary Affiliation (short), City, Country *

University of Toronto, Toronto, Canada

University of Gießen, Ge

Your e-mail address *

abc@gmail.com

titus.brinker@gmail.com

Title of your manuscript *

Provide the (draft) title of your manuscript.

A Medical Student–Delivered Smoking Prevention Program, Education Against Tobacco, for Secondary Schools in Germany: Randomized Controlled Trial

Article Preparation Status/Stage *

At which stage in your article preparation are you currently (at the time you fill in this form)

- ○ not submitted yet - in early draft status
- ○ not submitted yet - in late draft status, just before submission
- ○ submitted to a journal but not reviewed yet
- ○ submitted to a journal and after receiving initial reviewer comments
- ● submitted to a journal and accepted, but not published yet
- ○ published
- ○ Sonstiges:

Journal *

If you already know where you will submit this paper (or if it is already submitted), please provide the journal name (if it is not JMIR, provide the journal name under "other")

- ○ not submitted yet / unclear where I will submit this
- ● Journal of Medical Internet Research (JMIR)
- ○ Sonstiges:

Manuscript tracking number *

If this is a JMIR submission, please provide the manuscript tracking number under "other" (The ms tracking number can be found in the submission acknowledgement email, or when you login as author in JMIR. If the paper is already published in JMIR, then the ms tracking number is the four-digit number at the end of the DOI, to be found at the bottom of each published article in JMIR)

- ○ no ms number (yet) / not (yet) submitted to / published in JMIR
- ● Sonstiges: 7906

TITLE AND ABSTRACT

1a) TITLE: Identification as a randomized trial in the title

1a) Does your paper address CONSORT item 1a? *

I.e does the title contain the phrase "Randomized Controlled Trial"? (if not, explain the reason under "other")

- (selected) yes
- Sonstiges:

1a-i) Identify the mode of delivery in the title

Identify the mode of delivery. Preferably use “web-based” and/or “mobile” and/or “electronic game” in the title. Avoid ambiguous terms like “online”, “virtual”, “interactive”. Use “Internet-based” only if Intervention includes non-web-based Internet components (e.g. email), use “computer-based” or “electronic” only if offline products are used. Use “virtual” only in the context of “virtual reality” (3-D worlds). Use “online” only in the context of “online support groups”. Complement or substitute product names with broader terms for the class of products (such as “mobile” or “smart phone” instead of “iphone”), especially if the application runs on different platforms.

	1	2	3	4	5	
subitem not at all important	○	○	●	○	○	essential

Does your paper address subitem 1a-i? *

Copy and paste relevant sections from manuscript title (include quotes in quotation marks "like this" to indicate direct quotes from your manuscript), or elaborate on this item by providing additional information not in the ms, or briefly explain why the item is not applicable/relevant for your study

Yes: "Medical Student–Delivered"

1a-ii) Non-web-based components or important co-interventions in title

Mention non-web-based components or important co-interventions in title, if any (e.g., “with telephone support”).

	1	2	3	4	5	
subitem not at all important	○	●	○	○	○	essential

Does your paper address subitem 1a-ii?

Copy and paste relevant sections from manuscript title (include quotes in quotation marks "like this" to indicate direct quotes from your manuscript), or elaborate on this item by providing additional information not in the ms, or briefly explain why the item is not applicable/relevant for your study

It is not relevant for my study because there is only one intervention.

1a-iii) Primary condition or target group in the title

Mention primary condition or target group in the title, if any (e.g., “for children with Type I Diabetes”)
Example: A Web-based and Mobile Intervention with Telephone Support for Children with Type I Diabetes: Randomized Controlled Trial

	1	2	3	4	5	
subitem not at all important	○	○	○	●	○	essential

Does your paper address subitem 1a-iii? *

Copy and paste relevant sections from manuscript title (include quotes in quotation marks "like this" to indicate direct quotes from your manuscript), or elaborate on this item by providing additional information not in the ms, or briefly explain why the item is not applicable/relevant for your study

"for Secondary Schools in Germany"

1b) ABSTRACT: Structured summary of trial design, methods, results, and conclusions

NPT extension: Description of experimental treatment, comparator, care providers, centers, and blinding status.

1b-i) Key features/functionalities/components of the intervention and comparator in the METHODS section of the ABSTRACT

Mention key features/functionalities/components of the intervention and comparator in the abstract. If possible, also mention theories and principles used for designing the site. Keep in mind the needs of systematic reviewers and indexers by including important synonyms. (Note: Only report in the abstract what the main paper is reporting. If this information is missing from the main body of text, consider adding it)

	1	2	3	4	5	
subitem not at all important	○	○	●	○	○	essential

Does your paper address subitem 1b-i? *

Copy and paste relevant sections from the manuscript abstract (include quotes in quotation marks "like this" to indicate direct quotes from your manuscript), or elaborate on this item by providing additional information not in the ms, or briefly explain why the item is not applicable/relevant for your study

"of the optimized 2014 EAT curriculum
involving a photoaging software"

1b-ii) Level of human involvement in the METHODS section of the ABSTRACT

Clarify the level of human involvement in the abstract, e.g., use phrases like “fully automated” vs. “therapist/nurse/care provider/physician-assisted” (mention number and expertise of providers involved, if any). (Note: Only report in the abstract what the main paper is reporting. If this information is missing from the main body of text, consider adding it)

	1	2	3	4	5	
subitem not at all important	○	○	●	○	○	essential

Does your paper address subitem 1b-ii?

Copy and paste relevant sections from the manuscript abstract (include quotes in quotation marks "like this" to indicate direct quotes from your manuscript), or elaborate on this item by providing additional information not in the ms, or briefly explain why the item is not applicable/relevant for your study

1b-iii) Open vs. closed, web-based (self-assessment) vs. face-to-face assessments in the METHODS section of the ABSTRACT

Mention how participants were recruited (online vs. offline), e.g., from an open access website or from a clinic or a closed online user group (closed usergroup trial), and clarify if this was a purely web-based trial, or there were face-to-face components (as part of the intervention or for assessment). Clearly say if outcomes were self-assessed through questionnaires (as common in web-based trials). Note: In traditional offline trials, an open trial (open-label trial) is a type of clinical trial in which both the researchers and participants know which treatment is being administered. To avoid confusion, use “blinded” or “unblinded” to indicated the level of blinding instead of “open”, as “open” in web-based trials usually refers to “open access” (i.e. participants can self-enrol). (Note: Only report in the abstract what the main paper is reporting. If this information is missing from the main body of text, consider adding it)

	1	2	3	4	5	
subitem not at all important	○	○	○	○	○	essential

Does your paper address subitem 1b-iii?

Copy and paste relevant sections from the manuscript abstract (include quotes in quotation marks "like this" to indicate direct quotes from your manuscript), or elaborate on this item by providing additional information not in the ms, or briefly explain why the item is not applicable/relevant for your study

A randomized controlled trial was undertaken with 1504 adolescents from 9 German secondary schools, aged 11-15
years in grades 6-8, of which 718 (47.74%) were identifiable for the prospective sample at the 12-month follow-up.

1b-iv) RESULTS section in abstract must contain use data

Report number of participants enrolled/assessed in each group, the use/uptake of the intervention (e.g., attrition/adherence metrics, use over time, number of logins etc.), in addition to primary/secondary outcomes. (Note: Only report in the abstract what the main paper is reporting. If this information is missing from the main body of text, consider adding it)

	1	2	3	4	5	
subitem not at all important	○	●	○	○	○	essential

Does your paper address subitem 1b-iv?

Copy and paste relevant sections from the manuscript abstract (include quotes in quotation marks "like this" to indicate direct quotes from your manuscript), or elaborate on this item by providing additional information not in the ms, or briefly explain why the item is not applicable/relevant for your study

None of the effects were significant due to a high loss-to-follow-up effect (52.26%, 786/1504).

1b-v) CONCLUSIONS/DISCUSSION in abstract for negative trials

Conclusions/Discussions in abstract for negative trials: Discuss the primary outcome - if the trial is negative (primary outcome not changed), and the intervention was not used, discuss whether negative results are attributable to lack of uptake and discuss reasons. (Note: Only report in the abstract what the main paper is reporting. If this information is missing from the main body of text, consider adding it)

	1	2	3	4	5	
subitem not at all important	○	○	○	●	○	essential

Does your paper address subitem 1b-v?

Copy and paste relevant sections from the manuscript abstract (include quotes in quotation marks "like this" to indicate direct quotes from your manuscript), or elaborate on this item by providing additional information not in the ms, or briefly explain why the item is not applicable/relevant for your study

"None of the effects were significant due to a high loss-to-follow-up effect (52.26%, 786/1504). The intervention appears to prevent smoking, especially in females and students with a low educational background."

INTRODUCTION

2a) In INTRODUCTION: Scientific background and explanation of rationale

2a-i) Problem and the type of system/solution

Describe the problem and the type of system/solution that is object of the study: intended as stand-alone intervention vs. incorporated in broader health care program? Intended for a particular patient population? Goals of the intervention, e.g., being more cost-effective to other interventions, replace or complement other solutions? (Note: Details about the intervention are provided in “Methods” under 5)

	1	2	3	4	5	
subitem not at all important	○	●	○	○	○	essential

Does your paper address subitem 2a-i? *

Copy and paste relevant sections from the manuscript (include quotes in quotation marks "like this" to indicate direct quotes from your manuscript), or elaborate on this item by providing additional information not in the ms, or briefly explain why the item is not applicable/relevant for your study

Most smokers start smoking during their early adolescence with the idea that smoking is glamorous; the problems related to vascular disease, lung cancer, chronic pulmonary disease and cataracts are too far in the future to fathom [1]. After multiple failed quit attempts, however, many smokers end up with tobacco-related diseases that physicians are unable to cure [2]. Despite the fact that effectiveness of inpatient smoking cessation was demonstrated in major trials [3] and was implemented in guidelines of almost all medical specialties [4], research [illegible]

2a-ii) Scientific background, rationale: What is known about the (type of) system

Scientific background, rationale: What is known about the (type of) system that is the object of the study (be sure to discuss the use of similar systems for other conditions/diagnoses, if appropiate), motivation for the study, i.e. what are the reasons for and what is the context for this specific study, from which stakeholder viewpoint is the study performed, potential impact of findings [2]. Briefly justify the choice of the comparator.

1 2 3 4 5

subitem not at all important ○ ○ ○ ● ○ essential

Does your paper address subitem 2a-ii? *

Copy and paste relevant sections from the manuscript (include quotes in quotation marks "like this" to indicate direct quotes from your manuscript), or elaborate on this item by providing additional information not in the ms, or briefly explain why the item is not applicable/relevant for your study

Previous Research on Education Against Tobacco
The effectiveness of an old version of the EAT curriculum on reducing smoking prevalence among German adolescents has only been investigated with a quasi-experimental design that contained potential sources of bias [11,12]. However, the study showed a significant association for reducing the smoking prevalence of secondary school students in Germany at 6-month follow-up by motivating them to make a quit attempt (n=1474 students). After this first evaluation, the curriculum

2b) In INTRODUCTION: Specific objectives or hypotheses

Does your paper address CONSORT subitem 2b? *

Copy and paste relevant sections from the manuscript (include quotes in quotation marks "like this" to indicate direct quotes from your manuscript), or elaborate on this item by providing additional information not in the ms, or briefly explain why the item is not applicable/relevant for your study

The aim of this study was to provide the first randomized long-term evaluation of the optimized 2014 EAT curriculum involving a photoaging software for its effectiveness in reducing the smoking prevalence among 11-to-15-year-old pupils in German secondary schools.

METHODS

3a) Description of trial design (such as parallel, factorial) including allocation ratio

Does your paper address CONSORT subitem 3a? *

Copy and paste relevant sections from the manuscript (include quotes in quotation marks "like this" to indicate direct quotes from your manuscript), or elaborate on this item by providing additional information not in the ms, or briefly explain why the item is not applicable/relevant for your study

The survey was designed as a randomized controlled trial with three measurements (baseline, 6 months, and 12 months postintervention) [12]. The study period was October 2014 until March 2016. Participants in the two study groups (intervention and control) were questioned up to 2 weeks in advance of the intervention (t1), and 6 months (t2) and 12 months (t3) thereafter (Figure 1).
Randomization
In accordance with the recommendations of the Cochrane Analysis [20],

3b) Important changes to methods after trial commencement (such as eligibility criteria), with reasons

Does your paper address CONSORT subitem 3b? *

Copy and paste relevant sections from the manuscript (include quotes in quotation marks "like this" to indicate direct quotes from your manuscript), or elaborate on this item by providing additional information not in the ms, or briefly explain why the item is not applicable/relevant for your study

No, there were no changes to the original protocol.

3b-i) Bug fixes, Downtimes, Content Changes

Bug fixes, Downtimes, Content Changes: ehealth systems are often dynamic systems. A description of changes to methods therefore also includes important changes made on the intervention or comparator during the trial (e.g., major bug fixes or changes in the functionality or content) (5-iii) and other "unexpected events" that may have influenced study design such as staff changes, system failures/downtimes, etc. [2].

	1	2	3	4	5	
subitem not at all important	○	●	○	○	○	essential

Does your paper address subitem 3b-i?

Copy and paste relevant sections from the manuscript (include quotes in quotation marks "like this" to indicate direct quotes from your manuscript), or elaborate on this item by providing additional information not in the ms, or briefly explain why the item is not applicable/relevant for your study

This does not apply to our intervention. There were no downtimes.

4a) Eligibility criteria for participants

Does your paper address CONSORT subitem 4a? *

Copy and paste relevant sections from the manuscript (include quotes in quotation marks "like this" to indicate direct quotes from your manuscript), or elaborate on this item by providing additional information not in the ms, or briefly explain why the item is not applicable/relevant for your study

Participants
A total of 1504 eligible secondary school students from 74 classes (from 9 eligible schools) entered baseline data. All participants fulfilled the inclusion criteria. Students aged 10-15 years attending grades 6-8 of a secondary general, intermediate, grammar, or comprehensive school were eligible [12]. Baseline data were collected from October 2014 to March 2015. Follow-up data were collected from April 2015 to March 2016. A total of 718 participants provided data at both time points (t1 [illegible]

4a-i) Computer / Internet literacy

Computer / Internet literacy is often an implicit "de facto" eligibility criterion - this should be explicitly clarified.

	1	2	3	4	5	
subitem not at all important	●	○	○	○	○	essential

Does your paper address subitem 4a-i?

Copy and paste relevant sections from the manuscript (include quotes in quotation marks "like this" to indicate direct quotes from your manuscript), or elaborate on this item by providing additional information not in the ms, or briefly explain why the item is not applicable/relevant for your study

No, but it is irrelevant as this is very clear.

4a-ii) Open vs. closed, web-based vs. face-to-face assessments:

Open vs. closed, web-based vs. face-to-face assessments: Mention how participants were recruited (online vs. offline), e.g., from an open access website or from a clinic, and clarify if this was a purely web-based trial, or there were face-to-face components (as part of the intervention or for assessment), i.e., to what degree got the study team to know the participant. In online-only trials, clarify if participants were quasi-anonymous and whether having multiple identities was possible or whether technical or logistical measures (e.g., cookies, email confirmation, phone calls) were used to detect/prevent these.

	1	2	3	4	5	
subitem not at all important	○	●	○	○	○	essential

Does your paper address subitem 4a-ii? *

Copy and paste relevant sections from the manuscript (include quotes in quotation marks "like this" to indicate direct quotes from your manuscript), or elaborate on this item by providing additional information not in the ms, or briefly explain why the item is not applicable/relevant for your study

The intervention was the 2014 EAT curriculum, which consisted of two interactive 60-minute modules delivered by medical students from the University of Giessen. The medical students did not volunteer but had the duty to perform a school visit based on their participation in the EAT course. The app version of Smokerface was available at the time but was not advertised or mentioned to the students, to avoid contamination of the control group. The medical students received standardized training that lasted 45 minutes. Students were asked in advance to read [illegible]

4a-iii) Information giving during recruitment

Information given during recruitment. Specify how participants were briefed for recruitment and in the informed consent procedures (e.g., publish the informed consent documentation as appendix, see also item X26), as this information may have an effect on user self-selection, user expectation and may also bias results.

	1	2	3	4	5	
subitem not at all important	○	●	○	○	○	essential

Does your paper address subitem 4a-iii?

Copy and paste relevant sections from the manuscript (include quotes in quotation marks "like this" to indicate direct quotes from your manuscript), or elaborate on this item by providing additional information not in the ms, or briefly explain why the item is not applicable/relevant for your study

In accordance with Good Epidemiologic Practice guidelines, an ethics waiver and all legal permissions were obtained from the responsible institutions before data collection started. Written informed consent was obtained from all students and their parents. Teachers had to be present during the intervention and school personnel in charge of possible adverse health events were present in each school visited.

4b) Settings and locations where the data were collected

Does your paper address CONSORT subitem 4b? *

Copy and paste relevant sections from the manuscript (include quotes in quotation marks "like this" to indicate direct quotes from your manuscript), or elaborate on this item by providing additional information not in the ms, or briefly explain why the item is not applicable/relevant for your study

"Education Against Tobacco (EAT) is a multinational network of medical students who volunteer for school-based prevention in the classroom setting"

4b-i) Report if outcomes were (self-)assessed through online questionnaires

Clearly report if outcomes were (self-)assessed through online questionnaires (as common in web-based trials) or otherwise.

	1	2	3	4	5	
subitem not at all important	(•)	( )	( )	( )	( )	essential

Does your paper address subitem 4b-i? *

Copy and paste relevant sections from the manuscript (include quotes in quotation marks "like this" to indicate direct quotes from your manuscript), or elaborate on this item by providing additional information not in the ms, or briefly explain why the item is not applicable/relevant for your study

Yes. "Data were collected via a published questionnaire that was used in the previous investigation of the same age group, which was optimized by cognitive interviewing [11,39]. All items were based on three established studies declared to be high quality by the recent Cochrane Analysis and were either used in their original form or adapted to the specific circumstances of the recent study [20,40-42]. In addition to sociodemographic data (age, gender, school type), the questionnaire captured the smoking status of the school students concerning e- [illegible]

4b-ii) Report how institutional affiliations are displayed

Report how institutional affiliations are displayed to potential participants [on ehealth media], as affiliations with prestigious hospitals or universities may affect volunteer rates, use, and reactions with regards to an intervention.(Not a required item – describe only if this may bias results)

	1	2	3	4	5	
subitem not at all important	( )	(•)	( )	( )	( )	essential

Does your paper address subitem 4b-ii?

Copy and paste relevant sections from the manuscript (include quotes in quotation marks "like this" to indicate direct quotes from your manuscript), or elaborate on this item by providing additional information not in the ms, or briefly explain why the item is not applicable/relevant for your study

No, but this was not relevant to our study.

5) The interventions for each group with sufficient details to allow replication, including how and when they were actually administered

5-i) Mention names, credential, affiliations of the developers, sponsors, and owners

Mention names, credential, affiliations of the developers, sponsors, and owners [6] (if authors/evaluators are owners or developer of the software, this needs to be declared in a “Conflict of interest” section or mentioned elsewhere in the manuscript).

	1	2	3	4	5	
subitem not at all important	○	○	○	○	●	essential

Does your paper address subitem 5-i?

Copy and paste relevant sections from the manuscript (include quotes in quotation marks "like this" to indicate direct quotes from your manuscript), or elaborate on this item by providing additional information not in the ms, or briefly explain why the item is not applicable/relevant for your study

The link to the full curriculum is provided.

5-ii) Describe the history/development process

Describe the history/development process of the application and previous formative evaluations (e.g., focus groups, usability testing), as these will have an impact on adoption/use rates and help with interpreting results.

	1	2	3	4	5	
subitem not at all important	○	●	○	○	○	essential

Does your paper address subitem 5-ii?

Copy and paste relevant sections from the manuscript (include quotes in quotation marks "like this" to indicate direct quotes from your manuscript), or elaborate on this item by providing additional information not in the ms, or briefly explain why the item is not applicable/relevant for your study

The effectiveness of an old version of the EAT curriculum on reducing smoking prevalence among German adolescents has only been investigated with a quasi-experimental design that contained potential sources of bias [11,12]. However, the study showed a significant association for reducing the smoking prevalence of secondary school students in Germany at 6-month follow-up by motivating them to make a quit attempt (n=1474 students). After this first evaluation, the curriculum was optimized for students with a lower educational level by using [illegible]

5-iii) Revisions and updating

Revisions and updating. Clearly mention the date and/or version number of the application/intervention (and comparator, if applicable) evaluated, or describe whether the intervention underwent major changes during the evaluation process, or whether the development and/or content was “frozen” during the trial. Describe dynamic components such as news feeds or changing content which may have an impact on the replicability of the intervention (for unexpected events see item 3b).

	1	2	3	4	5	
subitem not at all important	○	●	○	○	○	essential

Does your paper address subitem 5-iii?

Copy and paste relevant sections from the manuscript (include quotes in quotation marks "like this" to indicate direct quotes from your manuscript), or elaborate on this item by providing additional information not in the ms, or briefly explain why the item is not applicable/relevant for your study

The effectiveness of an old version of the EAT curriculum on reducing smoking prevalence among German adolescents has only been investigated with a quasi-experimental design that contained potential sources of bias [11,12]. However, the study showed a significant association for reducing the smoking prevalence of secondary school students in Germany at 6-month follow-up by motivating them to make a quit attempt (n=1474 students). After this first evaluation, the curriculum was optimized for students with a lower educational level by using

5-iv) Quality assurance methods

Provide information on quality assurance methods to ensure accuracy and quality of information provided [1], if applicable.

	1	2	3	4	5	
subitem not at all important	○	●	○	○	○	essential

Does your paper address subitem 5-iv?

Copy and paste relevant sections from the manuscript (include quotes in quotation marks "like this" to indicate direct quotes from your manuscript), or elaborate on this item by providing additional information not in the ms, or briefly explain why the item is not applicable/relevant for your study

The medical students received standardized training that lasted 45 minutes. Students were asked in advance to read through the classroom curriculum and then met with an experienced medical student who went through all key procedures of the intervention.

5-v) Ensure replicability by publishing the source code, and/or providing screenshots/screen-capture video, and/or providing flowcharts of the algorithms used

Ensure replicability by publishing the source code, and/or providing screenshots/screen-capture video, and/or providing flowcharts of the algorithms used. Replicability (i.e., other researchers should in principle be able to replicate the study) is a hallmark of scientific reporting.

	1	2	3	4	5	
subitem not at all important	○	●	○	○	○	essential

Does your paper address subitem 5-v?

Copy and paste relevant sections from the manuscript (include quotes in quotation marks "like this" to indicate direct quotes from your manuscript), or elaborate on this item by providing additional information not in the ms, or briefly explain why the item is not applicable/relevant for your study

Photoaging desktop programs, in which an image is altered to predict future appearance, were effective in motivating 14-to-18-year-old females to quit smoking and increased the rate of quit attempts in 18-to-30-year-old young adults of both genders by 21% [34,35]. The broad availability of smartphones and adolescents' interest in their appearance [36] were harnessed to create the free 3D-photoaging smartphone app Smokerface [15] which animates the users' selfies and reacts to touch (Multimedia Appendix 1). This app is downloaded 200 times per day and

5-vi) Digital preservation

Digital preservation: Provide the URL of the application, but as the intervention is likely to change or disappear over the course of the years; also make sure the intervention is archived (Internet Archive, webcitation.org, and/or publishing the source code or screenshots/videos alongside the article). As pages behind login screens cannot be archived, consider creating demo pages which are accessible without login.

	1	2	3	4	5	
subitem not at all important	○	●	○	○	○	essential

Does your paper address subitem 5-vi?

Copy and paste relevant sections from the manuscript (include quotes in quotation marks "like this" to indicate direct quotes from your manuscript), or elaborate on this item by providing additional information not in the ms, or briefly explain why the item is not applicable/relevant for your study

Photoaging desktop programs, in which an image is altered to predict future appearance, were effective in motivating 14-to-18-year-old females to quit smoking and increased the rate of quit attempts in 18-to-30-year-old young adults of both genders by 21% [34,35]. The broad availability of smartphones and adolescents' interest in their appearance [36] were harnessed to create the free 3D-photoaging smartphone app Smokerface [15] which animates the users' selfies and reacts to touch (Multimedia Appendix 1). This app is downloaded 200 times per day and [illegible]

5-vii) Access

Access: Describe how participants accessed the application, in what setting/context, if they had to pay (or were paid) or not, whether they had to be a member of specific group. If known, describe how participants obtained "access to the platform and Internet" [1]. To ensure access for editors/reviewers/readers, consider to provide a "backdoor" login account or demo mode for reviewers/readers to explore the application (also important for archiving purposes, see vi).

	1	2	3	4	5	
subitem not at all important	○	●	○	○	○	essential

Does your paper address subitem 5-vii? *

Copy and paste relevant sections from the manuscript (include quotes in quotation marks "like this" to indicate direct quotes from your manuscript), or elaborate on this item by providing additional information not in the ms, or briefly explain why the item is not applicable/relevant for your study

Four Macbook Air (Apple, California) computers were purchased to run the photoaging intervention in four classes simultaneously, and to make sure that every single pupil got their face photoaged. Wireless Local Area Network (W-LAN) sticks were also purchased, as many schools did not have W-LAN at the time but the app had to communicate with servers to perform the morphing process (the current version of the app runs offline). Every student received the photoaging intervention; groups of five pupils were sent out to an external room where one medical [illegible]

5-viii) Mode of delivery, features/functionalities/components of the intervention and comparator, and the theoretical framework

Describe mode of delivery, features/functionalities/components of the intervention and comparator, and the theoretical framework [6] used to design them (instructional strategy [1], behaviour change techniques, persuasive features, etc., see e.g., [7, 8] for terminology). This includes an in-depth description of the content (including where it is coming from and who developed it) [1]," whether [and how] it is tailored to individual circumstances and allows users to track their progress and receive feedback" [6]. This also includes a description of communication delivery channels and – if computer-mediated communication is a component – whether communication was synchronous or asynchronous [6]. It also includes information on presentation strategies [1], including page design principles, average amount of text on pages, presence of hyperlinks to other resources, etc. [1].

1 2 3 4 5

subitem not at all important ◯ ◉ ◯ ◯ ◯ essential

Does your paper address subitem 5-viii? *

Copy and paste relevant sections from the manuscript (include quotes in quotation marks "like this" to indicate direct quotes from your manuscript), or elaborate on this item by providing additional information not in the ms, or briefly explain why the item is not applicable/relevant for your study

Four Macbook Air (Apple, California) computers were purchased to run the photoaging intervention in four classes simultaneously, and to make sure that every single pupil got their face photoaged. Wireless Local Area Network (W-LAN) sticks were also purchased, as many schools did not have W-LAN at the time but the app had to communicate with servers to perform the morphing process (the current version of the app runs offline). Every student received the photoaging intervention; groups of five pupils were sent out to an external room where one medical

5-ix) Describe use parameters

Describe use parameters (e.g., intended "doses" and optimal timing for use). Clarify what instructions or recommendations were given to the user, e.g., regarding timing, frequency, heaviness of use, if any, or was the intervention used ad libitum.

	1	2	3	4	5	
subitem not at all important	◯	◉	◯	◯	◯	essential

Does your paper address subitem 5-ix?

Copy and paste relevant sections from the manuscript (include quotes in quotation marks "like this" to indicate direct quotes from your manuscript), or elaborate on this item by providing additional information not in the ms, or briefly explain why the item is not applicable/relevant for your study

Four Macbook Air (Apple, California) computers were purchased to run the photoaging intervention in four classes simultaneously, and to make sure that every single pupil got their face photoaged. Wireless Local Area Network (W-LAN) sticks were also purchased, as many schools did not have W-LAN at the time but the app had to communicate with servers to perform the morphing process (the current version of the app runs offline). Every student received the photoaging intervention; groups of five pupils were sent out to an external room where one medical

5-x) Clarify the level of human involvement

Clarify the level of human involvement (care providers or health professionals, also technical assistance) in the e-intervention or as co-intervention (detail number and expertise of professionals involved, if any, as well as "type of assistance offered, the timing and frequency of the support, how it is initiated, and the medium by which the assistance is delivered". It may be necessary to distinguish between the level of human involvement required for the trial, and the level of human involvement required for a routine application outside of a RCT setting (discuss under item 21 – generalizability).

	1	2	3	4	5	
subitem not at all important	◯	◉	◯	◯	◯	essential

Does your paper address subitem 5-x?

Copy and paste relevant sections from the manuscript (include quotes in quotation marks "like this" to indicate direct quotes from your manuscript), or elaborate on this item by providing additional information not in the ms, or briefly explain why the item is not applicable/relevant for your study

The first part of the intervention was presented by two-to-six medical students and a patient with a tobacco-related disease to all pupils in a large room within the school. The presentation consisted of a PowerPoint (Microsoft; Redmond, WA, USA) presentation which aimed at addressing age-appropriate topics in an interactive manner to help the students reframe a positive nonsmoking image, which included: physical performance (with the example of famous German soccer players), saved money, addiction versus freedom, examples of tobacco [illegible]

5-xi) Report any prompts/reminders used

Report any prompts/reminders used: Clarify if there were prompts (letters, emails, phone calls, SMS) to use the application, what triggered them, frequency etc. It may be necessary to distinguish between the level of prompts/reminders required for the trial, and the level of prompts/reminders for a routine application outside of a RCT setting (discuss under item 21 – generalizability).

	1	2	3	4	5	
subitem not at all important	(•)	( )	( )	( )	( )	essential

Does your paper address subitem 5-xi? *

Copy and paste relevant sections from the manuscript (include quotes in quotation marks "like this" to indicate direct quotes from your manuscript), or elaborate on this item by providing additional information not in the ms, or briefly explain why the item is not applicable/relevant for your study

No, as there were no reminders.

5-xii) Describe any co-interventions (incl. training/support)

Describe any co-interventions (incl. training/support): Clearly state any interventions that are provided in addition to the targeted eHealth intervention, as ehealth intervention may not be designed as stand-alone intervention. This includes training sessions and support [1]. It may be necessary to distinguish between the level of training required for the trial, and the level of training for a routine application outside of a RCT setting (discuss under item 21 – generalizability.

	1	2	3	4	5	
subitem not at all important	(•)	( )	( )	( )	( )	essential

Does your paper address subitem 5-xii? *

Copy and paste relevant sections from the manuscript (include quotes in quotation marks "like this" to indicate direct quotes from your manuscript), or elaborate on this item by providing additional information not in the ms, or briefly explain why the item is not applicable/relevant for your study

The first part of the intervention was presented by two-to-six medical students and a patient with a tobacco-related disease to all pupils in a large room within the school. The presentation consisted of a PowerPoint (Microsoft; Redmond, WA, USA) presentation which aimed at addressing age-appropriate topics in an interactive manner to help the students reframe a positive nonsmoking image, which included: physical performance (with the example of famous German soccer players), saved money, addiction versus freedom, examples of tobacco [illegible]

6a) Completely defined pre-specified primary and secondary outcome measures, including how and when

they were assessed

Does your paper address CONSORT subitem 6a? *

Copy and paste relevant sections from the manuscript (include quotes in quotation marks "like this" to indicate direct quotes from your manuscript), or elaborate on this item by providing additional information not in the ms, or briefly explain why the item is not applicable/relevant for your study

The primary endpoint was the difference in smoking prevalence from t1 to t3 in the control group versus the difference from t1 to t3 in the intervention group. The differences in smoking behavior (smoking onset, quit attempts) between the two groups, as well as gender-specific effects, were studied as secondary outcomes.

6a-i) Online questionnaires: describe if they were validated for online use and apply CHERRIES items to describe how the questionnaires were designed/deployed

If outcomes were obtained through online questionnaires, describe if they were validated for online use and apply CHERRIES items to describe how the questionnaires were designed/deployed [9].

	1	2	3	4	5	
subitem not at all important	(•)	( )	( )	( )	( )	essential

Does your paper address subitem 6a-i?

Copy and paste relevant sections from manuscript text

No online questionnaires were used.

6a-ii) Describe whether and how “use” (including intensity of use/dosage) was defined/measured/monitored

Describe whether and how “use” (including intensity of use/dosage) was defined/measured/monitored (logins, logfile analysis, etc.). Use/adoption metrics are important process outcomes that should be reported in any ehealth trial.

	1	2	3	4	5	
subitem not at all important	(•)	( )	( )	( )	( )	essential

Does your paper address subitem 6a-ii?

Copy and paste relevant sections from manuscript text

Four Macbook Air (Apple, California) computers were purchased to run the photoaging intervention in four classes simultaneously, and to make sure that every single pupil got their face photoaged.

6a-iii) Describe whether, how, and when qualitative feedback from participants was obtained

Describe whether, how, and when qualitative feedback from participants was obtained (e.g., through emails, feedback forms, interviews, focus groups).

1 2 3 4 5

subitem not at all important ◉ ○ ○ ○ ○ essential

Does your paper address subitem 6a-iii?

Copy and paste relevant sections from manuscript text

Data were collected via a published questionnaire that was used in the previous investigation of the same age group, which was optimized by cognitive interviewing [11,39]. All items were based on three established studies declared to be high quality by the recent Cochrane Analysis and were either used in their original form or adapted to the specific circumstances of the recent study [20,40-42]. In addition to sociodemographic data (age, gender, school type), the questionnaire captured the smoking status of the school students concerning e-[illegible]

6b) Any changes to trial outcomes after the trial commenced, with reasons

Does your paper address CONSORT subitem 6b? *

Copy and paste relevant sections from the manuscript (include quotes in quotation marks "like this" to indicate direct quotes from your manuscript), or elaborate on this item by providing additional information not in the ms, or briefly explain why the item is not applicable/relevant for your study

No changes were made.

7a) How sample size was determined

NPT: When applicable, details of whether and how the clustering by care provides or centers was addressed

7a-i) Describe whether and how expected attrition was taken into account when calculating the sample size

Describe whether and how expected attrition was taken into account when calculating the sample size.

1 2 3 4 5

subitem not at all important ◉ ○ ○ ○ ○ essential

Does your paper address subitem 7a-i?

Copy and paste relevant sections from manuscript title (include quotes in quotation marks "like this" to indicate direct quotes from your manuscript), or elaborate on this item by providing additional information not in the ms, or briefly explain why the item is not applicable/relevant for your study

We just used the same sample size calculation as in our previous study but this did not apply due to an unexpectedly high loss to follow up: "None of the effects were significant due to a high loss-to-follow-up effect (52.26%, 786/1504). "

7b) When applicable, explanation of any interim analyses and stopping guidelines

Does your paper address CONSORT subitem 7b? *

Copy and paste relevant sections from the manuscript (include quotes in quotation marks "like this" to indicate direct quotes from your manuscript), or elaborate on this item by providing additional information not in the ms, or briefly explain why the item is not applicable/relevant for your study

Not applicable.

8a) Method used to generate the random allocation sequence

NPT: When applicable, how care providers were allocated to each trial group

Does your paper address CONSORT subitem 8a? *

Copy and paste relevant sections from the manuscript (include quotes in quotation marks "like this" to indicate direct quotes from your manuscript), or elaborate on this item by providing additional information not in the ms, or briefly explain why the item is not applicable/relevant for your study

In accordance with the recommendations of the Cochrane Analysis [20], randomization was externally and centrally performed by a statistician from the University of Frankfurt, Germany on the class level within each school via block randomization. Due to the fact that the statistician performed the randomization one school at a time, there was a slight imbalance between groups (40 intervention and 34 control classes).

8b) Type of randomisation; details of any restriction (such as blocking and block size)

Does your paper address CONSORT subitem 8b? *

Copy and paste relevant sections from the manuscript (include quotes in quotation marks "like this" to indicate direct quotes from your manuscript), or elaborate on this item by providing additional information not in the ms, or briefly explain why the item is not applicable/relevant for your study

In accordance with the recommendations of the Cochrane Analysis [20], randomization was externally and centrally performed by a statistician from the University of Frankfurt, Germany on the class level within each school via block randomization. Due to the fact that the statistician performed the randomization one school at a time, there was a slight imbalance between groups (40 intervention and 34 control classes).

9) Mechanism used to implement the random allocation sequence (such as sequentially numbered containers), describing any steps taken to conceal the sequence until interventions were assigned

Does your paper address CONSORT subitem 9? *

Copy and paste relevant sections from the manuscript (include quotes in quotation marks "like this" to indicate direct quotes from your manuscript), or elaborate on this item by providing additional information not in the ms, or briefly explain why the item is not applicable/relevant for your study

In accordance with the recommendations of the Cochrane Analysis [20], randomization was externally and centrally performed by a statistician from the University of Frankfurt, Germany on the class level within each school via block randomization. Due to the fact that the statistician performed the randomization one school at a time, there was a slight imbalance between groups (40 intervention and 34 control classes).

10) Who generated the random allocation sequence, who enrolled participants, and who assigned participants to interventions

Does your paper address CONSORT subitem 10? *

Copy and paste relevant sections from the manuscript (include quotes in quotation marks "like this" to indicate direct quotes from your manuscript), or elaborate on this item by providing additional information not in the ms, or briefly explain why the item is not applicable/relevant for your study

In accordance with the recommendations of the Cochrane Analysis [20], randomization was externally and centrally performed by a statistician from the University of Frankfurt, Germany on the class level within each school via block randomization. Due to the fact that the statistician performed the randomization one school at a time, there was a slight imbalance between groups (40 intervention and 34 control classes).

11a) If done, who was blinded after assignment to interventions (for example, participants, care providers, those assessing outcomes) and how

NPT: Whether or not administering co-interventions were blinded to group assignment

11a-i) Specify who was blinded, and who wasn't

Specify who was blinded, and who wasn't. Usually, in web-based trials it is not possible to blind the participants [1, 3] (this should be clearly acknowledged), but it may be possible to blind outcome assessors, those doing data analysis or those administering co-interventions (if any).

	1	2	3	4	5	
subitem not at all important	(•)	( )	( )	( )	( )	essential

Does your paper address subitem 11a-i? *

Copy and paste relevant sections from the manuscript (include quotes in quotation marks "like this" to indicate direct quotes from your manuscript), or elaborate on this item by providing additional information not in the ms, or briefly explain why the item is not applicable/relevant for your study

No blinded study.

11a-ii) Discuss e.g., whether participants knew which intervention was the "intervention of interest" and which one was the "comparator"

Informed consent procedures (4a-ii) can create biases and certain expectations - discuss e.g., whether participants knew which intervention was the "intervention of interest" and which one was the "comparator".

	1	2	3	4	5	
subitem not at all important	(•)	( )	( )	( )	( )	essential

Does your paper address subitem 11a-ii?

Copy and paste relevant sections from the manuscript (include quotes in quotation marks "like this" to indicate direct quotes from your manuscript), or elaborate on this item by providing additional information not in the ms, or briefly explain why the item is not applicable/relevant for your study

This is not specifically addressed.

11b) If relevant, description of the similarity of interventions

(this item is usually not relevant for ehealth trials as it refers to similarity of a placebo or sham intervention to a active medication/intervention)

Does your paper address CONSORT subitem 11b? *

Copy and paste relevant sections from the manuscript (include quotes in quotation marks "like this" to indicate direct quotes from your manuscript), or elaborate on this item by providing additional information not in the ms, or briefly explain why the item is not applicable/relevant for your study

Not relevant.

12a) Statistical methods used to compare groups for primary and secondary outcomes

NPT: When applicable, details of whether and how the clustering by care providers or centers was addressed

Does your paper address CONSORT subitem 12a? *

Copy and paste relevant sections from the manuscript (include quotes in quotation marks "like this" to indicate direct quotes from your manuscript), or elaborate on this item by providing additional information not in the ms, or briefly explain why the item is not applicable/relevant for your study

To examine baseline differences χ2tests, t-tests and Mann-Whitney U Tests were used (see Multimedia Appendix 2 for details). The effects of predictors (gender, culture, and social characteristics) on smoking behavior after 12 months (t3) were calculated by robust panel logistic regression analysis. The significance level was 5% for t-tests (double-sided) and 95% for CIs (double-sided). Statistical analyses were performed using SPSS Statistics Version 23 (IBM; Armong, USA) and STATA 14 (StataCorp; Texas, USA). The group allocation in the sample [illegible]

12a-i) Imputation techniques to deal with attrition / missing values

Imputation techniques to deal with attrition / missing values: Not all participants will use the intervention/comparator as intended and attrition is typically high in ehealth trials. Specify how participants who did not use the application or dropped out from the trial were treated in the statistical analysis (a complete case analysis is strongly discouraged, and simple imputation techniques such as LOCF may also be problematic [4]).

	1	2	3	4	5	
subitem not at all important	●	○	○	○	○	essential

Does your paper address subitem 12a-i? *

Copy and paste relevant sections from the manuscript (include quotes in quotation marks "like this" to indicate direct quotes from your manuscript), or elaborate on this item by providing additional information not in the ms, or briefly explain why the item is not applicable/relevant for your study

The participants who dropped out at follow-up (t3) were analyzed with logistic regression analysis and showed no systematic bias regarding the interaction between: study group and smoking status (P=0.520), study group and gender (P=0.131), study group and age (P=0.427), or study group and school type (grammar school vs lower educational school type; P=0.440). However, there was a systematic bias regarding gender as a main effect (odds ratio [OR]=0.511, 95% CI 0.412-0.633, P<0.001) meaning that more boys dropped out than girls. There was a [illegible]

12b) Methods for additional analyses, such as subgroup analyses and adjusted analyses

Does your paper address CONSORT subitem 12b? *

Copy and paste relevant sections from the manuscript (include quotes in quotation marks "like this" to indicate direct quotes from your manuscript), or elaborate on this item by providing additional information not in the ms, or briefly explain why the item is not applicable/relevant for your study

To examine baseline differences χ2tests, t-tests and Mann-Whitney U Tests were used (see Multimedia Appendix 2 for details). The effects of predictors (gender, culture, and social characteristics) on smoking behavior after 12 months (t3) were calculated by robust panel logistic regression analysis. The significance level was 5% for t-tests (double-sided) and 95% for CIs (double-sided). Statistical analyses were performed using SPSS Statistics Version 23 (IBM; Armong, USA) and STATA 14 (StataCorp; Texas, USA). The group allocation in the sample [illegible]

X26) REB/IRB Approval and Ethical Considerations [recommended as subheading under "Methods"] (not a CONSORT item)

X26-i) Comment on ethics committee approval

	1	2	3	4	5	
subitem not at all important	○	●	○	○	○	essential

Does your paper address subitem X26-i?

Copy and paste relevant sections from the manuscript (include quotes in quotation marks "like this" to indicate direct quotes from your manuscript), or elaborate on this item by providing additional information not in the ms, or briefly explain why the item is not applicable/relevant for your study

In accordance with Good Epidemiologic Practice guidelines, an ethics waiver and all legal permissions were obtained from the responsible institutions before data collection started.

x26-ii) Outline informed consent procedures

Outline informed consent procedures e.g., if consent was obtained offline or online (how? Checkbox, etc.?), and what information was provided (see 4a-ii). See [6] for some items to be included in informed consent documents.

	1	2	3	4	5	
subitem not at all important	○	○	○	●	○	essential

Does your paper address subitem X26-ii?

Copy and paste relevant sections from the manuscript (include quotes in quotation marks "like this" to indicate direct quotes from your manuscript), or elaborate on this item by providing additional information not in the ms, or briefly explain why the item is not applicable/relevant for your study

In accordance with Good Epidemiologic Practice guidelines, an ethics waiver and all legal permissions were obtained from the responsible institutions before data collection started. Written informed consent was obtained from all students and their parents. Teachers had to be present during the intervention and school personnel in charge of possible adverse health events were present in each school visited. No adverse events occurred.

X26-iii) Safety and security procedures

Safety and security procedures, incl. privacy considerations, and any steps taken to reduce the likelihood or detection of harm (e.g., education and training, availability of a hotline)

	1	2	3	4	5	
subitem not at all important	●	○	○	○	○	essential

Does your paper address subitem X26-iii?

Copy and paste relevant sections from the manuscript (include quotes in quotation marks "like this" to indicate direct quotes from your manuscript), or elaborate on this item by providing additional information

not in the ms, or briefly explain why the item is not applicable/relevant for your study

"In accordance with Good Epidemiologic Practice guidelines, an ethics waiver and all legal permissions were obtained from the responsible institutions before data collection started. Written informed consent was obtained from all students and their parents. Teachers had to be present during the intervention and school personnel in charge of possible adverse health events were present in each school visited. No adverse events occurred."

RESULTS

13a) For each group, the numbers of participants who were randomly assigned, received intended treatment, and were analysed for the primary outcome

NPT: The number of care providers or centers performing the intervention in each group and the number of patients treated by each care provider in each center

Does your paper address CONSORT subitem 13a? *

Copy and paste relevant sections from the manuscript (include quotes in quotation marks "like this" to indicate direct quotes from your manuscript), or elaborate on this item by providing additional information not in the ms, or briefly explain why the item is not applicable/relevant for your study

Analyses of the data were based on the originally assigned groups (see Table 1). There were 400 pupils in the intervention group and 318 pupils in the control group who had participated in the survey at both relevant time points that could be identified (baseline sample=1504; prospective sample=718 pupils; loss to follow-up=786 pupils).

13b) For each group, losses and exclusions after randomisation, together with reasons

Does your paper address CONSORT subitem 13b? (NOTE: Preferably, this is shown in a CONSORT flow diagram) *

Copy and paste relevant sections from the manuscript (include quotes in quotation marks "like this" to indicate direct quotes from your manuscript), or elaborate on this item by providing additional information not in the ms, or briefly explain why the item is not applicable/relevant for your study

Attrition Analysis
The participants who dropped out at follow-up (t3) were analyzed with logistic regression analysis and showed no systematic bias regarding the interaction between: study group and smoking status (P=0.520), study group and gender (P=0.131), study group and age (P=0.427), or study group and school type (grammar school vs lower educational school type; P=0.440). However, there was a systematic bias regarding gender as a main effect (odds ratio [OR]=0.511, 95% CI 0.412-0.633, [illegible]

13b-i) Attrition diagram

Strongly recommended: An attrition diagram (e.g., proportion of participants still logging in or using the intervention/comparator in each group plotted over time, similar to a survival curve) or other figures or tables demonstrating usage/dose/engagement.

	1	2	3	4	5	
subitem not at all important	○	●	○	○	○	essential

Does your paper address subitem 13b-i?

Copy and paste relevant sections from the manuscript or cite the figure number if applicable (include quotes in quotation marks "like this" to indicate direct quotes from your manuscript), or elaborate on this item by providing additional information not in the ms, or briefly explain why the item is not applicable/relevant for your study

Attrition Analysis
The participants who dropped out at follow-up (t3) were analyzed with logistic regression analysis and showed no systematic bias regarding the interaction between: study group and smoking status (P=0.520), study group and gender (P=0.131), study group and age (P=0.427), or study group and school type (grammar school vs lower educational school type; P=0.440). However, there was a systematic bias regarding gender as a main effect (odds ratio [OR]=0.511, 95% CI 0.412-0.633, [illegible]

14a) Dates defining the periods of recruitment and follow-up

Does your paper address CONSORT subitem 14a? *

Copy and paste relevant sections from the manuscript (include quotes in quotation marks "like this" to indicate direct quotes from your manuscript), or elaborate on this item by providing additional information not in the ms, or briefly explain why the item is not applicable/relevant for your study

Baseline data were collected from October 2014 to March 2015. Follow-up data were collected from April 2015 to March 2016. A total of 718 participants provided data at both time points (t1 and t3) that was used for primary endpoint analyses. The loss to follow-up effect was 52.26% (786/1504) due to problems with the identifier code (see Discussion section).

14a-i) Indicate if critical “secular events” fell into the study period

Indicate if critical “secular events” fell into the study period, e.g., significant changes in Internet resources available or “changes in computer hardware or Internet delivery resources”

	1	2	3	4	5	
subitem not at all important	○	○	○	○	○	essential

Does your paper address subitem 14a-i?

Copy and paste relevant sections from the manuscript (include quotes in quotation marks "like this" to indicate direct quotes from your manuscript), or elaborate on this item by providing additional information not in the ms, or briefly explain why the item is not applicable/relevant for your study

The loss to follow-up effect was 52.26% (786/1504) due to problems with the identifier code (see Discussion section).

14b) Why the trial ended or was stopped (early)

Does your paper address CONSORT subitem 14b? *

Copy and paste relevant sections from the manuscript (include quotes in quotation marks "like this" to indicate direct quotes from your manuscript), or elaborate on this item by providing additional information not in the ms, or briefly explain why the item is not applicable/relevant for your study

No, it was not stopped.

15) A table showing baseline demographic and clinical characteristics for each group

NPT: When applicable, a description of care providers (case volume, qualification, expertise, etc.) and centers (volume) in each group

Does your paper address CONSORT subitem 15? *

Copy and paste relevant sections from the manuscript (include quotes in quotation marks "like this" to indicate direct quotes from your manuscript), or elaborate on this item by providing additional information not in the ms, or briefly explain why the item is not applicable/relevant for your study

Yes, multimedia appendix 2.

15-i) Report demographics associated with digital divide issues

In ehealth trials it is particularly important to report demographics associated with digital divide issues, such as age, education, gender, social-economic status, computer/Internet/ehealth literacy of the participants, if known.

	1	2	3	4	5	
subitem not at all important	(•)	( )	( )	( )	( )	essential

Does your paper address subitem 15-i? *

Copy and paste relevant sections from the manuscript (include quotes in quotation marks "like this" to indicate direct quotes from your manuscript), or elaborate on this item by providing additional information not in the ms, or briefly explain why the item is not applicable/relevant for your study

No, not relevant for our trial.

16) For each group, number of participants (denominator) included in each analysis and whether the analysis was by original assigned groups

16-i) Report multiple “denominators” and provide definitions

Report multiple “denominators” and provide definitions: Report N’s (and effect sizes) “across a range of study participation [and use] thresholds” [1], e.g., N exposed, N consented, N used more than x times, N used more than y weeks, N participants “used” the intervention/comparator at specific pre-defined time points of interest (in absolute and relative numbers per group). Always clearly define “use” of the intervention.

	1	2	3	4	5	
subitem not at all important	(•)	( )	( )	( )	( )	essential

Does your paper address subitem 16-i? *

Copy and paste relevant sections from the manuscript (include quotes in quotation marks "like this" to indicate direct quotes from your manuscript), or elaborate on this item by providing additional information not in the ms, or briefly explain why the item is not applicable/relevant for your study

Analyses of the data were based on the originally assigned groups (see Table 1). There were 400 pupils in the intervention group and 318 pupils in the control group who had participated in the survey at both relevant time points that could be identified (baseline sample=1504; prospective sample=718 pupils; loss to follow-up=786 pupils).

16-ii) Primary analysis should be intent-to-treat

Primary analysis should be intent-to-treat, secondary analyses could include comparing only “users”, with the appropriate caveats that this is no longer a randomized sample (see 18-i).

	1	2	3	4	5	
subitem not at all important	(•)	( )	( )	( )	( )	essential

Does your paper address subitem 16-ii?

Copy and paste relevant sections from the manuscript (include quotes in quotation marks "like this" to indicate direct quotes from your manuscript), or elaborate on this item by providing additional information not in the ms, or briefly explain why the item is not applicable/relevant for your study

17a) For each primary and secondary outcome, results for each group, and the estimated effect size and its precision (such as 95% confidence interval)

Does your paper address CONSORT subitem 17a? *

Copy and paste relevant sections from the manuscript (include quotes in quotation marks "like this" to indicate direct quotes from your manuscript), or elaborate on this item by providing additional information not in the ms, or briefly explain why the item is not applicable/relevant for your study

Primary Endpoint
There was no significant effect for the defined primary endpoint (OR=0.74; 95% CI 0.21-2.56; P=0.63) calculated with the prospective sample of 718 participants (Table 2). The percentage of students who claimed to be smokers increased from 3.1% (t1) to 7.2% (t3) in the control group, but the increase was less dramatic in the intervention group (3% [t1] to 5.4% [t3]).
Secondary Outcomes

17a-i) Presentation of process outcomes such as metrics of use and intensity of use

In addition to primary/secondary (clinical) outcomes, the presentation of process outcomes such as metrics of use and intensity of use (dose, exposure) and their operational definitions is critical. This does not only refer to metrics of attrition (13-b) (often a binary variable), but also to more continuous exposure metrics such as “average session length”. These must be accompanied by a technical description how a metric like a “session” is defined (e.g., timeout after idle time) [1] (report under item 6a).

	1	2	3	4	5	
subitem not at all important	(•)	( )	( )	( )	( )	essential

Does your paper address subitem 17a-i?

Copy and paste relevant sections from the manuscript (include quotes in quotation marks "like this" to indicate direct quotes from your manuscript), or elaborate on this item by providing additional information not in the ms, or briefly explain why the item is not applicable/relevant for your study

17b) For binary outcomes, presentation of both absolute and relative effect sizes is recommended

Does your paper address CONSORT subitem 17b? *

Copy and paste relevant sections from the manuscript (include quotes in quotation marks "like this" to indicate direct quotes from your manuscript), or elaborate on this item by providing additional information not in the ms, or briefly explain why the item is not applicable/relevant for your study

Yes, in the tables both is visible.

18) Results of any other analyses performed, including subgroup analyses and adjusted analyses, distinguishing pre-specified from exploratory

Does your paper address CONSORT subitem 18? *

Copy and paste relevant sections from the manuscript (include quotes in quotation marks "like this" to indicate direct quotes from your manuscript), or elaborate on this item by providing additional information

not in the ms, or briefly explain why the item is not applicable/relevant for your study

Yes.

18-i) Subgroup analysis of comparing only users

A subgroup analysis of comparing only users is not uncommon in ehealth trials, but if done, it must be stressed that this is a self-selected sample and no longer an unbiased sample from a randomized trial (see 16-iii).

	1	2	3	4	5	
subitem not at all important	●	○	○	○	○	essential

Does your paper address subitem 18-i?

Copy and paste relevant sections from the manuscript (include quotes in quotation marks "like this" to indicate direct quotes from your manuscript), or elaborate on this item by providing additional information not in the ms, or briefly explain why the item is not applicable/relevant for your study

19) All important harms or unintended effects in each group

(for specific guidance see CONSORT for harms)

Does your paper address CONSORT subitem 19? *

Copy and paste relevant sections from the manuscript (include quotes in quotation marks "like this" to indicate direct quotes from your manuscript), or elaborate on this item by providing additional information not in the ms, or briefly explain why the item is not applicable/relevant for your study

No adverse events occurred.

19-i) Include privacy breaches, technical problems

Include privacy breaches, technical problems. This does not only include physical “harm” to participants, but also incidents such as perceived or real privacy breaches [1], technical problems, and other unexpected/unintended incidents. “Unintended effects” also includes unintended positive effects [2].

	1	2	3	4	5	
subitem not at all important	●	○	○	○	○	essential

Does your paper address subitem 19-i?

Copy and paste relevant sections from the manuscript (include quotes in quotation marks "like this" to indicate direct quotes from your manuscript), or elaborate on this item by providing additional information not in the ms, or briefly explain why the item is not applicable/relevant for your study

19-ii) Include qualitative feedback from participants or observations from staff/researchers

Include qualitative feedback from participants or observations from staff/researchers, if available, on strengths and shortcomings of the application, especially if they point to unintended/unexpected effects or uses. This includes (if available) reasons for why people did or did not use the application as intended by the developers.

	1	2	3	4	5	
subitem not at all important	(•)	( )	( )	( )	( )	essential

Does your paper address subitem 19-ii?

Copy and paste relevant sections from the manuscript (include quotes in quotation marks "like this" to indicate direct quotes from your manuscript), or elaborate on this item by providing additional information not in the ms, or briefly explain why the item is not applicable/relevant for your study

DISCUSSION

22) Interpretation consistent with results, balancing benefits and harms, and considering other relevant evidence

NPT: In addition, take into account the choice of the comparator, lack of or partial blinding, and unequal expertise of care providers or centers in each group

22-i) Restate study questions and summarize the answers suggested by the data, starting with primary outcomes and process outcomes (use)

Restate study questions and summarize the answers suggested by the data, starting with primary outcomes and process outcomes (use).

	1	2	3	4	5	
subitem not at all important	( )	( )	( )	(•)	( )	essential

Does your paper address subitem 22-i? *

Copy and paste relevant sections from the manuscript (include quotes in quotation marks "like this" to indicate direct quotes from your manuscript), or elaborate on this item by providing additional information

not in the ms, or briefly explain why the item is not applicable/relevant for your study

This is the first long-term evaluation of a photoaging intervention to prevent smoking and the first completed randomized trial on medical-student-delivered school-based tobacco prevention to date [16]. The present study suggests that photoaging is effective at preventing smoking onset, especially in female students (NNT=24) and students with a low educational (NNT=30) or migrational backgrounds (NNT=44).

22-ii) Highlight unanswered new questions, suggest future research

Highlight unanswered new questions, suggest future research.

	1	2	3	4	5	
subitem not at all important	○	○	●	○	○	essential

Does your paper address subitem 22-ii?

Copy and paste relevant sections from the manuscript (include quotes in quotation marks "like this" to indicate direct quotes from your manuscript), or elaborate on this item by providing additional information not in the ms, or briefly explain why the item is not applicable/relevant for your study

Further research and long-term evaluation in sufficiently powered trials, as well as new ways of implementation, are needed to further evaluate and optimize our program.

20) Trial limitations, addressing sources of potential bias, imprecision, and, if relevant, multiplicity of analyses

20-i) Typical limitations in ehealth trials

Typical limitations in ehealth trials: Participants in ehealth trials are rarely blinded. Ehealth trials often look at a multiplicity of outcomes, increasing risk for a Type I error. Discuss biases due to non-use of the intervention/usability issues, biases through informed consent procedures, unexpected events.

	1	2	3	4	5	
subitem not at all important	○	○	○	○	○	essential

Does your paper address subitem 20-i? *

Copy and paste relevant sections from the manuscript (include quotes in quotation marks "like this" to indicate direct quotes from your manuscript), or elaborate on this item by providing additional information not in the ms, or briefly explain why the item is not applicable/relevant for your study

Limitations
While the groups were successfully randomized and provided comparable baseline data, an unusually high loss-to-follow-up effect led to no significant results to report. However, the descriptive data collected at three different time points and effect size estimates, such as number needed to treat (which is widely accepted as an indicator for clinical relevance), allowed for data interpretation [46]. The photoaging software became available as an app during the study period and was advertised [illegible]

21) Generalisability (external validity, applicability) of the trial findings

NPT: External validity of the trial findings according to the intervention, comparators, patients, and care providers or centers involved in the trial

21-i) Generalizability to other populations

Generalizability to other populations: In particular, discuss generalizability to a general Internet population, outside of a RCT setting, and general patient population, including applicability of the study results for other organizations

	1	2	3	4	5	
subitem not at all important	○	○	●	○	○	essential

Does your paper address subitem 21-i?

Copy and paste relevant sections from the manuscript (include quotes in quotation marks "like this" to indicate direct quotes from your manuscript), or elaborate on this item by providing additional information not in the ms, or briefly explain why the item is not applicable/relevant for your study

Interpretation
Available cross-sectional data reveals that photoaging interventions are effective in motivating 14-18-year-old female smokers to make a quit attempt [35], so it was hypothesized that secondary preventive effects would be present in the sample, which was not the case. However, the intervention showed a smaller NNT for females versus males in preventing smoking onset (NNT=24 for females vs 207 for males) and the uptake of smoking for females in the intervention group was lower [illegible]

21-ii) Discuss if there were elements in the RCT that would be different in a routine application setting

Discuss if there were elements in the RCT that would be different in a routine application setting (e.g., prompts/reminders, more human involvement, training sessions or other co-interventions) and what impact the omission of these elements could have on use, adoption, or outcomes if the intervention is applied outside of a RCT setting.

	1	2	3	4	5	
subitem not at all important	○	○	○	○	○	essential

Does your paper address subitem 21-ii?

Copy and paste relevant sections from the manuscript (include quotes in quotation marks "like this" to indicate direct quotes from your manuscript), or elaborate on this item by providing additional information not in the ms, or briefly explain why the item is not applicable/relevant for your study

OTHER INFORMATION

23) Registration number and name of trial registry

Does your paper address CONSORT subitem 23? *

Copy and paste relevant sections from the manuscript (include quotes in quotation marks "like this" to indicate direct quotes from your manuscript), or elaborate on this item by providing additional information not in the ms, or briefly explain why the item is not applicable/relevant for your study

"In accordance with Good Epidemiologic Practice guidelines, an ethics waiver and all legal permissions were obtained from the responsible institutions before data collection started. "

The study was not considered a clinical trial at the time and therefore was not registered outside of our ethics committee.

24) Where the full trial protocol can be accessed, if available

Does your paper address CONSORT subitem 24? *

Cite a Multimedia Appendix, other reference, or copy and paste relevant sections from the manuscript (include quotes in quotation marks "like this" to indicate direct quotes from your manuscript), or elaborate on this item by providing additional information not in the ms, or briefly explain why the item is not applicable/relevant for your study

No, not applicable / relevant.

25) Sources of funding and other support (such as supply of drugs), role of funders

Does your paper address CONSORT subitem 25? *

Copy and paste relevant sections from the manuscript (include quotes in quotation marks "like this" to indicate direct quotes from your manuscript), or elaborate on this item by providing additional information not in the ms, or briefly explain why the item is not applicable/relevant for your study

All funding is declared.

X27) Conflicts of Interest (not a CONSORT item)

X27-i) State the relation of the study team towards the system being evaluated

In addition to the usual declaration of interests (financial or otherwise), also state the relation of the study team towards the system being evaluated, i.e., state if the authors/evaluators are distinct from or identical with the developers/sponsors of the intervention.

	1	2	3	4	5	
subitem not at all important	○	○	○	○	○	essential

Does your paper address subitem X27-i?
Copy and paste relevant sections from the manuscript (include quotes in quotation marks "like this" to indicate direct quotes from your manuscript), or elaborate on this item by providing additional information not in the ms, or briefly explain why the item is not applicable/relevant for your study

About the CONSORT EHEALTH checklist

As a result of using this checklist, did you make changes in your manuscript? *

- ( ) yes, major changes
- ( ) yes, minor changes
- (x) no

What were the most important changes you made as a result of using this checklist?

How much time did you spend on going through the checklist INCLUDING making changes in your manuscript *

60 minutes.

As a result of using this checklist, do you think your manuscript has improved? *

- ( ) yes
- (x) no
- ( ) Sonstiges:

Would you like to become involved in the CONSORT EHEALTH group?
This would involve for example becoming involved in participating in a workshop and writing an "Explanation and Elaboration" document

- ( ) yes
- ( ) no
- ( ) Sonstiges:

Any other comments or questions on CONSORT EHEALTH

STOP - Save this form as PDF before you click submit

To generate a record that you filled in this form, we recommend to generate a PDF of this page (on a Mac, simply select "print" and then select "print as PDF") before you submit it.

When you submit your (revised) paper to JMIR, please upload the PDF as supplementary file.

Don't worry if some text in the textboxes is cut off, as we still have the complete information in our database. Thank you!

Final step: Click submit !

Click submit so we have your answers in our database!

Senden

Geben Sie niemals Passwörter über Google Formulare weiter.

Bereitgestellt von

Dieser Inhalt wurde nicht von Google erstellt und wird von Google auch nicht unterstützt.

Missbrauch melden - Nutzungsbedingungen - Zusätzliche Bestimmungen
